# Clinical trial landscape of cell therapy for spinal cord injury: from integrated practices to future developments

**DOI:** 10.1186/s12916-026-05078-2

**Published:** 2026-07-20

**Authors:** Jun Kang, Senyu Yao, Songfu Zou, Yangfan Yu, Xu Huang, Ziqi Xie, Yaobang Lin, Bin Liu, Mao Pang, Limin Rong

**Affiliations:** 1 Department of Spine Surgery, The Third Affiliated Hospital of Sun Yat-Sen University, Guangzhou, 510630 China; 2 Key Laboratory for Quality Research and Evaluation of Cell Products, National Medical Products Administration (NMPA), The Third Affiliated Hospital of Sun Yat-Sen University, Guangzhou, 510630 China; 3 Guangdong Engineering Technology Research Center of Minimally Invasive Spine Surgery, The Third Affiliated Hospital of Sun Yat-Sen University, Guangzhou, 510630 China

**Keywords:** Spinal cord injury, Cell therapy, Clinical trial, Safety profile, Efficacy outcome, Research trends, Regenerative medicine

## Abstract

**Introduction:**

Spinal cord injury (SCI) precipitates a multiphasic secondary injury cascade that establishes a hostile, inhibitory microenvironment, rendering the condition refractory to conventional surgical stabilization and rehabilitation. While cell-based therapies offer promise for neural reconstruction, their clinical translation is impeded by protocol heterogeneity and fragmented safety data. To address this, we mapped clinical trials for SCI to quantify patient demographic parameters, identify lineage-specific adverse-event patterns, evaluate objective motor and sensory efficacy outcomes, and analyze methodological trial designs to formulate concrete structural recommendations for future advanced-phase trials.

**Methods:**

We analyzed clinical trials from the Web of Science Core Collection (SCIE and ESCI) published since 2005. The dataset comprised 116 eligible studies involving patients with SCI receiving cellular therapies with reported safety outcomes. We employed a dual-method approach combining manual extraction of clinical characteristics (demographics, interventions, adverse events, efficacy outcomes) with quantitative data analysis. Statistical associations between therapeutic variables (cell type, route, dosage) and safety profiles were evaluated using Fisher’s exact test.

**Results:**

The clinical landscape is predominantly defined by early-phase (Phase 1: 59.5%), single-arm investigations (63.8%) utilizing autologous bone marrow-derived cells. Reflecting a cautious paradigm to minimize severe complications, patient selection frequently targeted the hemodynamically stable chronic phase (58.3%) and thoracic SCI (21.6%). Safety analyses revealed lineage-specific profiles: mesenchymal stromal cells were significantly associated with transient fever (*P * = 0.038), whereas intrathecal administration correlated with procedural symptoms such as headache (*P* < 0.001). The observation of higher systemic adverse event rates in low-dose cohorts was likely confounded by the mandatory concurrent immunosuppressive regimens required for specific allogeneic lineages, rather than the absolute cell dose. Regarding therapeutic efficacy, outcomes were critically influenced by the chronological phase of injury. Patients treated in the acute or subacute phases exhibited higher rates of neurological improvement, though distinguishing this from spontaneous recovery remains challenging, whereas chronic phase interventions demonstrated more limited primary sensorimotor gains. Additionally, intrathecal administration showed an advantage in preserving sensory pathways due to minimized structural disruption, while dose requirements could not be generalized and varied fundamentally based on specific cellular mechanisms of action.

**Discussion:**

While the baseline safety of cellular transplantation for spinal cord injury is established, clinical translation remains hindered by methodological heterogeneity, imprecise patient stratification, and a reliance on single-arm trial designs. To navigate this translational bottleneck, future investigations should adopt multi-tiered methodological frameworks. First, study designs should transition toward controlled protocols, utilizing crossover designs for chronic cohorts and matched historical or synthetic controls for acute and subacute phases. Concurrently, patient selection must evolve from broad clinical grading to advanced stratification. Integrating biomarkers, electrophysiology, and imaging to objectively quantify tissue sparing can better identify responsive subgroups, thereby improving trial efficiency and accelerating clinical translation. Beyond cohort refinement, intervention parameters, specifically dosage and delivery routes, should be individualized according to cell lineage, as adverse events associate more with intrinsic cellular biology and procedural invasiveness. Furthermore, isolating the true therapeutic effect requires the standardization and reporting of confounding variables, such as immunosuppressive regimens and physical rehabilitation. Finally, by separating shorter-term efficacy measurements from long-term safety registries, the field can facilitate a more reliable and objective clinical translation.

**Supplementary Information:**

The online version contains supplementary material available at https://doi.org/10.1186/s12916-026-05078-2.

## Introduction

Spinal cord injury (SCI) constitutes a serious neurological condition of the central nervous system, clinically manifesting as lifelong disability characterized not only by motor paralysis but also by spasticity, sensory deficits, and severe autonomic dysfunction, including bowel and bladder impairment [1]. Despite advancements in preventive medicine, the global burden of SCI remains substantial and follows an escalating trajectory. Our recent epidemiological analysis identified a significant upward trend in incidence, prevalence, and years lived with disability (YLDs) from 1990 to 2019 [2]. In 2021 alone, an estimated 574,502 new cases were reported globally, corresponding to an age-standardized incidence of 7.1 per 100,000 individuals[3]. The epidemiological profile of SCI now exhibits a bimodal distribution. Particularly in industrialized nations, there is a rapidly growing incidence among the elderly, primarily driven by fall-related injuries. Regardless of the age of onset, the associated burden extends beyond physical morbidity, imposing a severe socioeconomic toll driven by the exigencies of lifelong care [4, 5]. The clinical refractoriness of SCI stems from its complex pathophysiology: the primary mechanical trauma precipitates a multiphasic secondary injury cascade, characterized by a deleterious synergy of neuroinflammation, oxidative stress, and blood-spinal cord barrier disruption [6–8]. This cascade leads to progressive neuronal and axonal loss, accompanied by the formation of a dense scar. Together, these events establish a hostile and inhibitory microenvironment that acts as both a physical and biochemical barrier to axonal regeneration [9–12].

Current clinical management of SCI relies heavily on prompt acute management and rehabilitation, which are vital for maximizing functional independence and preventing complications. Standard acute interventions, specifically early surgical decompression and hemodynamic augmentation, are critical for biomechanical stabilization and the mitigation of the secondary injury cascade. Notably, while high-dose methylprednisolone protocols were historically utilized, they are no longer recommended as a routine standard of care in recent global guidelines due to a lack of definitive efficacy and an increased risk of adverse events [13–15]. However, these conventional strategies are fundamentally limited by their inability to bridge neuroanatomical discontinuities or reverse established functional deficits [16]. Consequently, physical rehabilitation remains the primary, yet often insufficient, modality for maximizing residual function [17]. Although emerging neurotechnologies, such as brain-computer interfaces (BCIs) and epidural electrical stimulation (EES), have demonstrated potential in restoring motor control, these modalities function primarily as functional bypasses rather than agents of biological repair [18–21]. The incapacity of both conventional standards and engineered surrogates to reconstruct damaged neural architecture underscores a persistent therapeutic void, one that cell-based therapies are specifically theoretically situated to address [22].

In response to this clinical imperative, cell-based therapies have emerged as a pivotal frontier in regenerative medicine, operating through two principal paradigms: intrinsic neuronal reconstruction and extrinsic environmental modulation [23]. The former, driven primarily by neural stem/progenitor cells, aims to physically replace lost circuitry, whereas the latter utilizes olfactory ensheathing cells (OECs), mesenchymal stem cells (MSCs), and other lineages to ameliorate the inhibitory microenvironment and support endogenous regeneration [24, 25]. While recent large-scale reviews have significantly advanced the field by aggregating patient-level data to evaluate general cellular efficacy, several methodological gaps remain. Building upon these foundations, the present study addresses these gaps through three distinct strategic refinements [26–29]. Primarily, moving beyond descriptive safety summaries, we employ quantitative statistical tests to directly correlate specific regimen parameters, including cellular lineage and administration route, with the incidence of distinct adverse events [23, 30]. Furthermore, our efficacy analysis expands upon previous comparisons by utilizing visualizations to stratify neurological recovery across multiple variables [30]. Crucially, we evaluate the clinical landscape from a holistic trial design perspective to identify systemic translational bottlenecks. Thus, this unified and statistically driven synthesis provides a necessary framework to navigate the multidimensional heterogeneity of current clinical practices.

Therefore, rather than imposing a traditional quantitative synthesis upon highly disparate data, the present study aims to construct a comprehensive clinical landscape of cellular therapy for SCI. By integrating a systematic review of clinical practices with a quantitative landscape analysis, we mapped clinical trials to quantify specific patient demographic parameters and analyze methodological trial designs. We specifically evaluate the variability in cellular sources, dosages, and administration routes to propose standardized clinical baselines. Crucially, this analysis extends to a precise assessment of both safety and therapeutic efficacy. We correlate specific regimen parameters with the incidence of adverse events to define lineage-specific safety risks, while simultaneously quantifying objective motor and sensory score improvements to characterize observed therapeutic outcomes of distinct cellular grafts. Ultimately, by synthesizing evidence from both published literature and active clinical trial registries, this article outlines the requirements for future advanced-phase trials regarding double blind randomized designs and the strict standardization of concurrent immunosuppressive protocols and physical rehabilitation.

## Methods

### Study design and search strategy

To ensure a rigorous and reproducible methodology, this study adhered to the reporting standards outlined in the Preferred Reporting Items for Systematic Reviews and Meta-Analyses (PRISMA) guidelines, although the primary objective was a comprehensive landscape analysis rather than a traditional meta-analysis [31]. We performed a systematic literature search using the Web of Science Core Collection, encompassing the Science Citation Index Expanded (SCIE) and Emerging Sources Citation Index (ESCI) to ensure the inclusion of peer-reviewed scientific publications. Given that the clinical translation of cell therapies for spinal cord injury largely emerged in the mid-2000s, with prior literature predominantly consisting of sporadic case reports, the retrieval period was defined from January 1, 2005, to December 1, 2025. The search strategy employed a combination of keywords related to “Spinal cord injury,” “Cell therapy,” and “Clinical trial,” restricted to English-language publications; the detailed search string is provided in Supplementary Table 1 [32]. To mitigate the risk of publication bias and ensure comprehensive coverage, we further manually screened the reference lists of relevant systematic reviews and meta-analyses identified during the search. Subsequently, eligible studies found through this ancillary search were cross-referenced with our primary dataset and included if they met the selection criteria and were indexed in SCIE or ESCI.

### Inclusion and exclusion criteria

Eligibility criteria: Studies were selected based on the PICOS framework:

1. Participants (P): Human subjects diagnosed with spinal cord injury.
2. Interventions (I): Administration of any type of cell therapy or cell-derived products (e.g., exosomes/extracellular vesicles).
3. Comparisons (C): Studies were included regardless of the presence or type of a control group (e.g., placebo, standard of care, or no control).
4. Outcomes (O): Studies must report safety-related outcomes, including Adverse Events (AEs) or Serious Adverse Events (SAEs).
5. Study Design (S): Original clinical research (articles or research letters) and clinical trial protocols.

Exclusion criteria were as follows: (1) Animal studies or in vitro experiments; (2) Publications with data that were relevant but unextractable; (3) Case reports (defined as a sample size of fewer than 2 subjects), reviews, editorials, meeting abstracts, surveys, or patents; (4) Duplicate publications utilizing the same dataset; and (5) Studies involving patients with other confounding severe comorbidities.

### Data screening and management

Upon retrieving the bibliographic records, all files were exported in plain text format and managed using Zotero software to facilitate efficient duplicate removal and citation management. The screening process was conducted in two stages. Initially, titles and abstracts were screened to exclude clearly irrelevant records. Potentially eligible articles were then subjected to a full-text review to confirm final inclusion based on the predefined criteria. Two trained investigators (J.K. and S.Z.) independently screened the literature and extracted data, after which the selection results were cross-checked to ensure consistency. Any discrepancies that arose during this process were resolved through discussion or consultation with a corresponding author to reach a consensus.

### Data extraction and standardization

Data extraction was conducted using a dual-method approach to balance clinical detail with macroscopic bibliometric trends.

Manual Extraction for Clinical Characteristics: For clinical analysis, investigators manually extracted and cross-validated data across five domains. In cases of missing data, we attempted to contact the corresponding authors; however, some datasets remained incomplete.

1. Study Characteristics: Study design, clinical phase, sample size, and follow-up duration.
2. Patient Demographics: Sex, age, injury severity (AIS grade), injury chronicity (acute/subacute/chronic), and neurological level of injury.
3. Intervention Details: Cell source, cell type, route of administration, dosage, and frequency of delivery.
4. Safety Outcomes: Reported AEs and SAEs derived from clinical symptoms, laboratory tests, and imaging assessments.
5. Efficacy Outcomes: Objective neurological recovery metrics, primarily focusing on transitions in the AIS grade, changes in associated motor and sensory scores, radiological imaging findings, electrophysiological parameters, and multidimensional functional independence measures.

Automated Extraction for Bibliometric Analysis: To address the challenges of high-volume analysis and minimize human error in statistical calculations, we employed a multi-tool strategy to extract the fifth category of data: bibliometric metadata (titles, authors, publication dates, journals, institutions, countries/regions, keywords, citations, and references). These data were processed using VOSviewer (Version 1.6.20; Leiden University) and the R-based Bibliometrix package.

Prior to analysis, we performed rigorous data cleaning. This involved verifying authors with identical names by cross-referencing ORCID and institutional affiliations, as well as standardizing institution names to merge abbreviations with full names [33].

### Statistical analysis and visualization

Descriptive statistics and visualization techniques were employed to map the research landscape. For continuous demographic variables, specifically patient age, we utilized a meta-analytical approach to calculate the pooled mean with 95% confidence intervals using a random-effects model, thereby providing a statistically robust estimate of the population baseline despite the study not being a traditional efficacy meta-analysis. For the comparison of categorical variables, Fisher’s exact test was employed to calculate P-values, with statistical significance defined as *P* < 0.05. Regarding visualization, GraphPad Prism was utilized to generate stacked bar charts and donut charts for publication trends and distribution analysis. Meanwhile, R software via the ggplot2 package was utilized to create bubble charts, heatmaps, and dumbbell charts for multidimensional data representation. Furthermore, ScimagoGraphica was employed to visualize geospatial networks of country-level collaborations, whereas VOSviewer was used to construct network visualization maps for author productivity and keyword co-occurrence analysis.

## Results

### Literature search

**Fig. 1:**
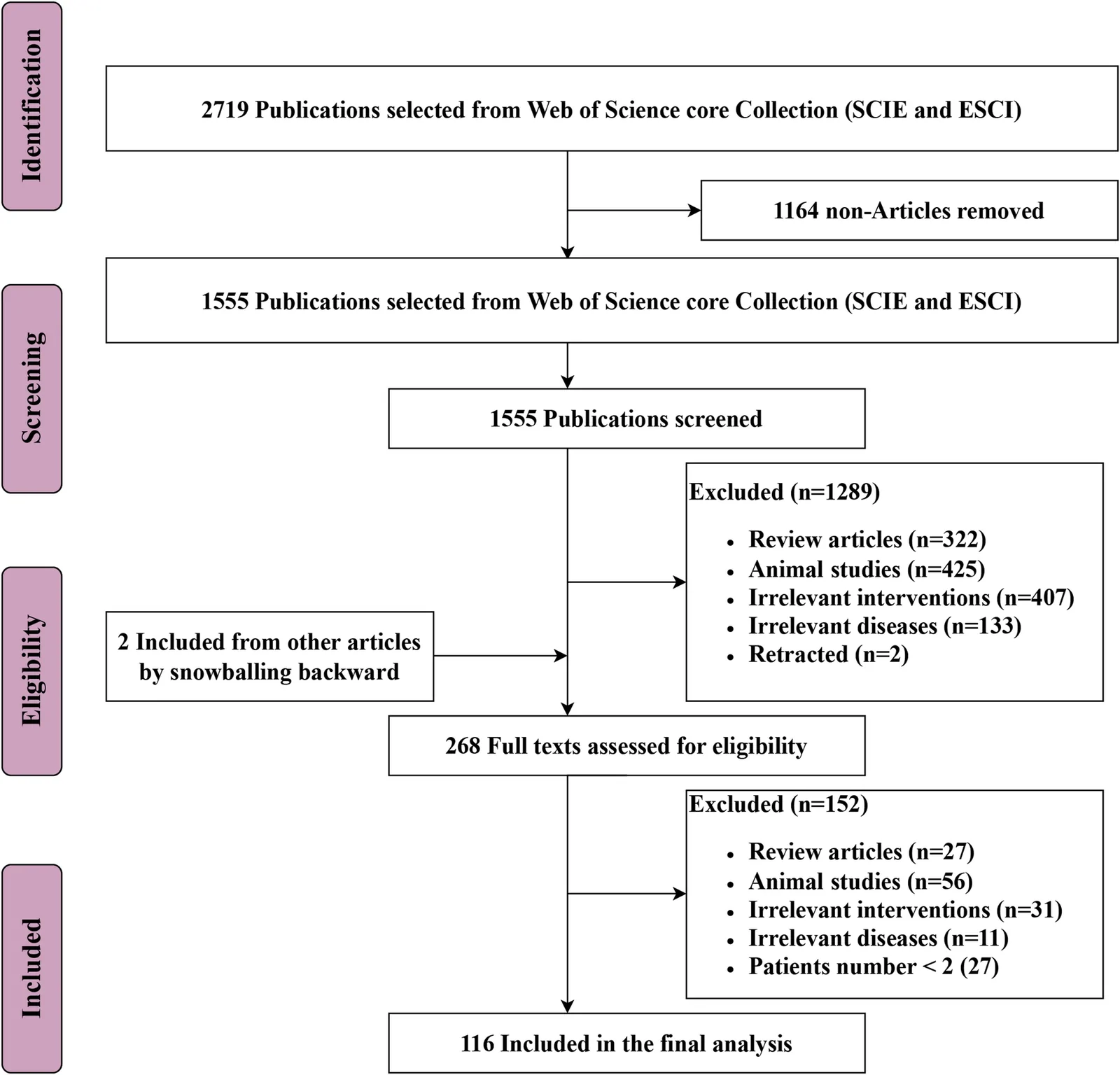
PRISMA Flow Diagram Illustrating the Literature Search and Study Selection Process. Abbreviations: ESCI, Emerging Sources Citation Index; PRISMA, Preferred Reporting Items for Systematic Reviews and Meta-Analyses; SCI, spinal cord injury; SCIE, Science Citation Index Expanded

Our literature search within the Web of Science Core Collection (SCIE and ESCI) initially identified a total of 2,719 publications. Following the removal of 1,164 non-primary research articles, 1,555 publications were deemed eligible for the initial screening phase. Through a preliminary review of titles and abstracts, we excluded 1,289 articles that did not meet the inclusion criteria. Concurrently, we identified 2 additional relevant studies by employing the backward snowballing technique on references from other articles. Consequently, a total of 268 full texts were assessed for eligibility. During this comprehensive review, 152 studies were further excluded for various reasons, including being review articles (*n* = 27), animal studies (*n* = 56), involving irrelevant interventions (*n* = 31), focusing on irrelevant diseases (*n* = 11), or having a patient cohort of fewer than two (*n* = 27). Ultimately, 116 studies satisfied all criteria and were included in the final analysis (Fig. 1). Detailed information regarding the key characteristics and outcome indicators of all included studies is systematically summarized in Supplementary Tables 2–4.

### Temporal trends and participant characteristics

**Fig. 2:**
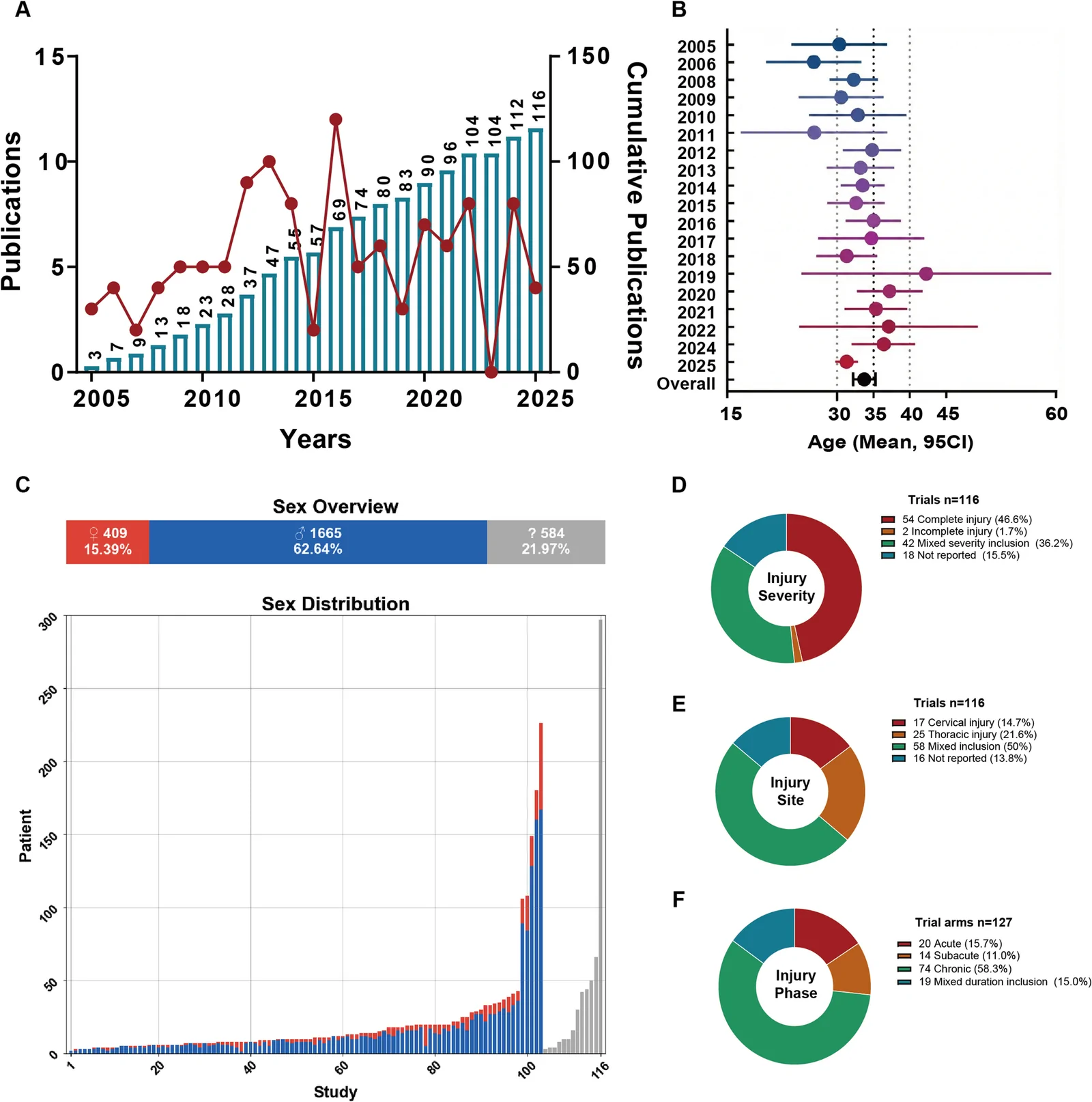
Temporal Trends and Clinical Characteristics of the Included Studies. **A **Annual volume of publications related to cell therapy for spinal cord injury. **B** Trends in the average age of study participants. **C** Gender distribution of the enrolled cohorts. **D** Classification of injury severity based on the AIS grades. **E** Distribution of the levels of injury. **F** Stratification of intervention phases. Abbreviations: AIS, ASIA Impairment Scale; ASIA, American Spinal Injury Association; SCI, spinal cord injury. Abbreviations: AIS, ASIA Impairment Scale; ASIA, American Spinal Injury Association; SCI, spinal cord injury

Quantitative analysis of the annual publication volume revealed a fluctuating upward trajectory over the study period, reaching a peak in 2016 with 12 publications (Fig. 2A). However, a sharp decline was observed subsequently, with zero studies published in 2023. This cessation of published results coincides with the global disruptions caused by the COVID-19 pandemic. Considering that cell therapy mandates strict regulatory oversight and must be administered within qualified medical centers, the pandemic-related lockdowns likely severely hampered the execution of such trials [34–36]. Furthermore, the inherent mobility limitations of patients with SCI, combined with restrictions on public transportation, presumably impeded patient recruitment and follow-up during this period [37, 38].

Regarding participant demographics, the study cohorts were predominantly composed of young adults. However, analysis indicates a gradual upward trend in the average age of enrolled patients, rising from approximately 30.3 years in the 2005 studies to 36.4 years in 2024 trials (Fig. 2B). In terms of gender distribution, male participants constituted the majority of the population (*n* = 1665, 62.64%), compared to female participants (Fig. 2C). This observation is consistent with established epidemiological data regarding the higher incidence and prevalence of SCI among males [39, 40].

### Injury characteristics

An assessment of injury severity demonstrated that the majority of clinical trials strictly focused on patients with complete SCI. Specifically, 46.6% of studies restricted enrollment exclusively to patients classified as AIS grade A, whereas only 1.7% involved only incomplete SCI corresponding to AIS grades B-E. The remaining trials either utilized mixed severity inclusion criteria (36.2%, enrolling both complete and incomplete SCI) or failed to clearly report this parameter (15.5%) (Fig. 2D).

Regarding the neurological level of injury, we analyzed the inclusion criteria at the trial level to understand investigator priorities. 50% of trials enrolled a combination of patients with cervical, thoracic, or lumbar SCI within the same study (Mixed neurological level inclusion). Among the trials that strictly restricted enrollment to a single region (cervical, thoracic, lumbar), the thoracic segment was the most frequently targeted site, accounting for 21.6% of all interventions (Fig. 2E), followed by cervical SCI (14.7%). Notably, only 13.8% of the included studies completely failed to report the injury level (Not reported). The slight preference for thoracic over cervical SCI in trials restricted to a single spinal region deviates somewhat from established epidemiological patterns, where the incidence of cervical versus thoracolumbar SCI is typically distributed in a roughly 50:50 ratio [39, 41, 42]. This discrepancy between clinical trial landscapes and real-world prevalence may reflect investigator caution. Patients with cervical SCI often present with significant instability in basic vital signs, including respiratory and hemodynamic fluctuations [43]. Furthermore, the administration of cell therapy via in situ delivery carries a potentially elevated risk of intraoperative complications when performed at the cervical level compared to the thoracic spine [44].

Finally, regarding the timing of intervention, the data showed that 58.3% of trial arms enrolled participants in the chronic phase of injury, significantly outnumbering those in the acute and subacute phases (Fig. 2F). This preference appears to be a strategic response to the complex challenges associated with the acute phase [13]. Clinically, patients in the acute post-injury period often exhibit severe physiological instability following trauma and primary surgical stabilization. The invasive nature of cell transplantation procedures, particularly those involving autologous harvesting and grafting, may further compromise these vulnerable patients [22]. Moreover, the neuroinflammatory response present in the acute injury microenvironment poses a hostile barrier to cell survival, thereby making the chronic phase a more viable target for cell transplantation efficacy [45].

### Characterization of delivery, biological sources, and therapeutic protocols

**Fig. 3:**
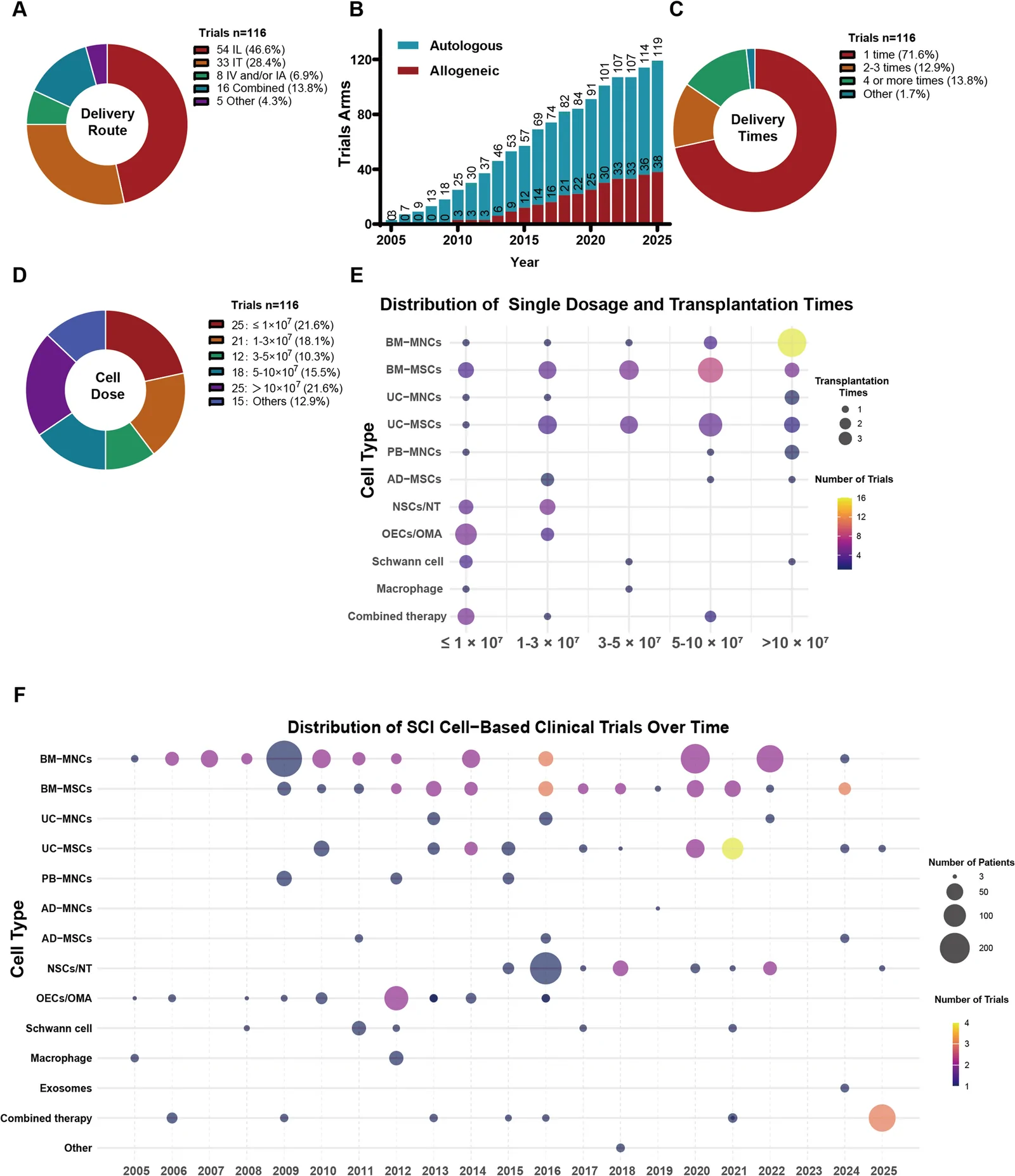
Landscape of Delivery Strategies, Dosing Regimens, and Temporal Evolution of Cell Sources. **A** Proportions of different cell delivery routes utilized in clinical trials. **B** Distribution of cell sources categorized by autologous and allogeneic origins. **C** Percentage of trials employing single versus multiple administration frequencies. **D** Global distribution of cell dosages across analyzed studies. **E** Stratification of dosage levels and administration frequencies by specific cell types. **F** Temporal trends and evolution of cell type application from 2005 to 2025. Abbreviations: IL, intralesional; IT, intrathecal; MNCs, mononuclear cells; MSCs, mesenchymal stem cells; NSCs, neural stem cells; NT, neural tissue; OECs, olfactory ensheathing cells; OMA, olfactory mucosa autograft; UC, umbilical cord

To elucidate the operational frameworks of cell therapy, we comprehensively analyzed the administration routes and biological sources utilized across the study landscape (Fig. 3). In terms of delivery modalities, local administration strategies predominated, driven by the clinical imperative to maximize cellular retention and bioavailability at the lesion site. Specifically, intralesional (IL) injection and intrathecal (IT) administration emerged as the primary approaches, accounting for 46.6% and 28.4% of the trials, respectively (Fig. 3A). Regarding cell origin, autologous transplantation, defined as the utilization of cells sourced directly from the patient receiving the treatment, has remained the cornerstone of clinical interventions since its inception in 2005, representing the majority with 81 trial arms. Conversely, allogeneic transplantation, which first appeared in the clinical arena in 2007, has been utilized in 38 trial arms to date, reflecting a more cautious adoption trajectory (Fig. 3B).

Given the inherent risks of immune rejection associated with allogeneic grafting, we further evaluated the concurrent use of immunosuppressive regimens (Supplementary Table 5). Notably, the application of immunosuppression exhibited a highly lineage-dependent pattern. Among the 38 allogeneic studies, explicit immunosuppressive protocols (frequently utilizing tacrolimus alone or in combination with mycophenolate mofetil) were systematically employed in 10 trials. These trials predominantly involved neural stem cells (NSCs) and oligodendrocyte progenitor cells (OPCs). In contrast, the majority of trials utilizing mesenchymal stromal cells (MSCs) or olfactory ensheathing cells (OECs) omitted exogenous immunosuppressants, a clinical decision likely predicated on the well-documented low immunogenicity and intrinsic immunomodulatory properties of these specific cell populations [46, 47]. Regarding the safety profile of these regimens, the immunosuppressive protocols were generally well-tolerated. Most studies reported no severe adverse events (SAEs) directly attributable to the drugs. Commonly reported adverse events were mild to moderate, primarily including hypomagnesemia, nausea, and opportunistic bacterial infections. However, the sporadic occurrence of severe complications, such as sepsis, underscores the necessity of rigorous patient monitoring when immunosuppression is mandated for graft survival.

We further evaluated the temporal and quantitative aspects of therapeutic protocols. An assessment of administration frequency indicated a prevailing preference for single-dose protocols, which constituted 71.6% of the analyzed trials (Fig. 3C). Although emerging evidence suggests that repetitive dosing may enhance therapeutic efficacy, the predominance of single-dose regimens likely reflects a prioritization of safety profiles and risk mitigation in clinical settings [48]. Furthermore, the overall distribution of cell dosages across the landscape appeared relatively balanced (Fig. 3D). However, a stratified analysis revealed distinct heterogeneity in dosing regimens contingent upon cell type (Fig. 3E). Specifically, bone marrow-derived MSCs were frequently administered at moderate to high dosages. A parallel trend was observed in umbilical cord-derived MSCs (UC-MSCs). This dosing strategy is supported by an extensive body of evidence confirming the robust safety profile of MSCs. Broad meta-analyses have established that MSC therapies are not associated with serious adverse events or long-term toxicity, while systematic reviews focused on UC-MSCs specifically report an absence of tumor formation or cell rejection [49–51]. Within the specific context of SCI, current evidence further corroborates that both MSCs and UC-MSCs are well-tolerated, even in high-dose or multi-dose protocols, due to their favorable immunomodulatory characteristics [26, 48. In contrast, neural-lineage cells, including NSCs and Schwann cells, as well as combined therapeutic approaches, were predominantly administered at lower absolute dosages and with fewer intervention frequencies when compared to MSCs and mononuclear cells (MNCs).

### Temporal evolution and future trends in cell types

Longitudinal analysis highlighted the temporal evolution of cell selection and research intensity (Fig. 3F). Bone marrow-derived cells have demonstrated sustained utility since 2005, accumulating the largest volume of studies and substantial sample sizes. While UC-MSCs and adipose-derived MSCs were introduced later, they have garnered increasing attention in recent years, paralleled by a rise in study quantity. Notably, the application of NSCs/NT expanded significantly following 2015, marking a shift towards neural-specific regeneration strategies. Detailed characterization of these NSC-based interventions (*n* = 10) revealed an equal reliance on primary fetal CNS tissue-derived cells (*n* = 5) and established neural cell lines (*n* = 5). Conversely, early investigative hotspots such as macrophages and OECs have seen a decline in recent focus. Furthermore, combined therapeutic strategies represent an emerging and rapidly developing frontier, evidenced by the publication of three related studies in 2025 alone. Among the 9 studies investigating cellular co-transplantation, the combination of MSCs and Schwann cells was the most frequently utilized (*n* = 5). Other innovative approaches included the combined delivery of MSCs with OECs (*n* = 1), OECs with Schwann cells (*n* = 1), MNCs with Schwann cells (*n* = 1), and MNCs with NSCs (*n* = 1). This trajectory indicates a strategic transition towards integrated multimodal treatments.

### Evaluation of safety profiles and adverse event reporting

**Fig. 4:**
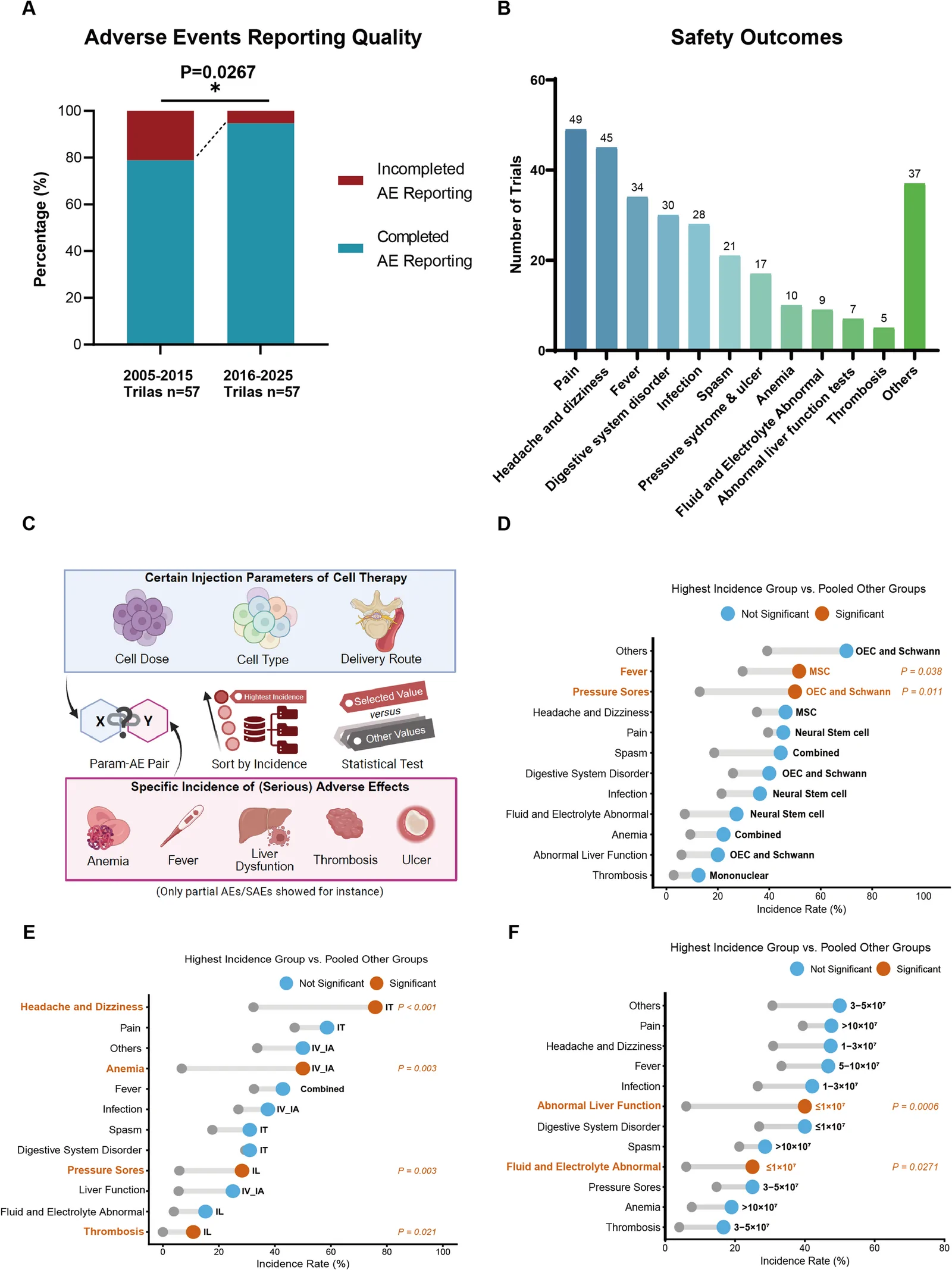
Overview of Adverse Event Reporting Quality and Safety Profile Analysis in Clinical Trials. **A** Evolution of adverse event (AE) reporting quality compared between the 2005–2015 and 2016–2025 cohorts. **B** Spectrum and frequency of specific safety outcomes. The presented frequencies indicate the proportion of included trials in which each respective adverse event was documented at least once. **C** Schematic diagram illustrating the analytical workflow for evaluating the associations between key interventional parameters (cell type, delivery route, and cell dose) and the specific incidence of adverse events. **D**–**F** Dumbbell plot analyses illustrating the associations between specific adverse events and clinical variables, including (**D**) cell types, (**E**) delivery routes, and (**F**) cell dosages. Blue dots indicate non-significant differences, while orange dots denote statistically significant associations (P < 0.05) between the highest incidence group and the pooled other groups. Abbreviations: AE, adverse event; MSC, mesenchymal stem cell; OEC, olfactory ensheathing cell; NSC, neural stem cell; IT, intrathecal; IV, intravenous; IA, intra-arterial; IL, intralesional

We conducted a comprehensive analysis of safety outcomes across 114 studies, excluding two protocols, to assess the evolution of reporting quality and the specific landscape of adverse events (AEs). Our longitudinal analysis of reporting quality revealed a significant improvement over time. In the earlier cohort spanning 2005 to 2015 (*n* = 57), research focus was predominantly confined to SAEs, resulting in 12 studies (21.1%) failing to provide a complete account of transient or symptomatic AEs. In contrast, the subsequent decade (2016–2025; *n* = 57) demonstrated a marked enhancement in rigor, with only 3 studies (5.3%) lacking detailed AE documentation (Fig. 4A, *P* = 0.0267). This temporal shift indicates a growing consensus within the field to prioritize comprehensive safety monitoring, extending beyond SAEs to capture the full spectrum of patient responses [30].

To characterize the specific safety risks associated with cell therapy, we synthesized data from the 99 trials that provided detailed AE reporting. Pain and headache/dizziness were the most prevalent complications, reported in 49 and 45 trials, respectively (Fig. 4B). Notably, our analysis highlights that AEs following cell therapy for SCI are systemic rather than localized solely to the nervous system. Documented events spanned the digestive system, respiratory and urinary systems (manifesting as infections), motor system (spasms), and the hematologic system (anemia and thrombosis). Furthermore, metabolic perturbations, including abnormal liver function and fluid/electrolyte imbalances, were observed. Within the category of “Others,” trials reported complications such as cerebrospinal fluid leakage, syringomyelia, transient deterioration of motor or sensory function, and allergic reactions. Serious adverse events, though less frequent, included critical conditions such as pulmonary embolism, aseptic meningitis, subarachnoid hemorrhage, and osteomyelitis.

We subsequently performed analyses to determine how different therapeutic variables influence safety profiles. To identify specific risk factors, we statistically compared the subgroup exhibiting the highest incidence of a given adverse event against a pooled cohort comprising all other respective subgroups (Fig. 4D-F). Regarding cell types, trials using MSCs more frequently reported fever than trials using non-MSC products (Fig. 4D, P **= 0.038**). This phenomenon is likely attributable to the immunomodulatory properties of MSCs, which can activate the host innate immune system and trigger a transient release of pro-inflammatory cytokines, such as IL-1β, IL-6, and TNF-α [52, 53]. Conversely, compared to the aggregate of all other cell types, OECs and Schwann cells showed a statistical association with pressure sores (*P* = 0.011). From a clinical perspective, these specific cell lineages and their respective harvesting procedures, such as endoscopic mucosal harvest, lack a direct mechanistic link to the development of pressure sores. This statistical correlation may be attributed to heterogeneous reporting standards across the included trials. Specifically, localized surgical wound complications, including skin lesions at the site of a peripheral nerve biopsy, may have been categorized as pressure sores in the primary publications.

The route of administration also played a distinct role in shaping the AE spectrum (Fig. 4E). IT administration was strongly associated with headache and dizziness (*P* < 0.001), presumably driven by post-lumbar puncture intracranial hypotension [54, 55].In the intravenous/intra-arterial (IV/IA) group, anemia was significantly more common compared to all localized delivery routes combined (*P* = 0.003). This phenomenon likely stems from the systemic nature of these administration routes, which maximizes the interface between the graft and the host’s circulatory and immune systems. Such systemic exposure can trigger immune-mediated complications, including transient hemolysis or immune-mediated interference with erythropoiesis, mechanisms well-recognized in other forms of systemic cell transplantation [56, 57]. Meanwhile, the IL administration cohort exhibited a higher statistical incidence of thrombosis relative to the pooled other administration routes (*P* = 0.021). However, this association is highly susceptible to confounding by the timing of the intervention. A detailed sub-analysis revealed that thrombosis was disproportionately reported in acute-phase trials (occurring in 18.8% [3/16] of acute trials versus only 3.1% [2/65] of chronic trials). Therefore, rather than being an iatrogenic consequence of the IL procedure itself, this finding heavily reflects the inherently high risk of thromboembolism characteristic of the acute phase of SCI [58]. To further validate these findings, we performed a lineage-specific subgroup analysis within the MSC cohort (Supplementary Fig. 1). The results were highly consistent with the aggregate data: IT administration of MSCs was significantly linked to headache and dizziness (*P* = 0.015), whereas the IV/IA route was primarily associated with anemia (*P* = 0.004). This internal consistency underscores that the observed toxicity profiles are primarily driven by the modality of delivery rather than the biological properties of the MSCs themselves.

Finally, we evaluated the impact of dosing strategies on safety outcomes. Contrary to the assumption that higher doses correlate with increased toxicity, we observed that the low-dose group was associated with a higher incidence of abnormal liver function (*P* = 0.0006) and fluid/electrolyte disturbances (*P* = 0.0271)(Fig. 4F). This observation is likely confounded by cell lineage and concurrent therapeutic protocols. Specifically, modalities characterized by intensive protocol requirements, such as NSCs, were frequently administered at lower dosages. To further investigate this confounding effect, we evaluated the dosage distribution stratified by immunosuppression status across allogeneic trials (Supplementary Fig. 2). Among the 10 trials utilizing NSCs, all of which mandated concurrent immunosuppression, 100% of the studies with reported dosage parameters (9 out of 9) administered cell doses of ≤ 3 × 10^7^. In contrast, among the 28 allogeneic trials utilizing non-NSC lineages without exogenous immunosuppressants, only 50% (12 out of 24) of the reported dosages fell within this low-dose range, with the remaining 50% utilizing dosages > 3 × 10^7^. Consequently, the higher incidence of systemic adverse events observed in this low-dose cohort is likely associated with these concurrent pharmacological regimens, most notably the mandatory systemic immunosuppression, rather than the absolute cell dose itself.

### Evaluation of clinical efficacy, functional independence, and secondary complication metrics and patient-level neurological recovery profiles

**Fig. 5:**
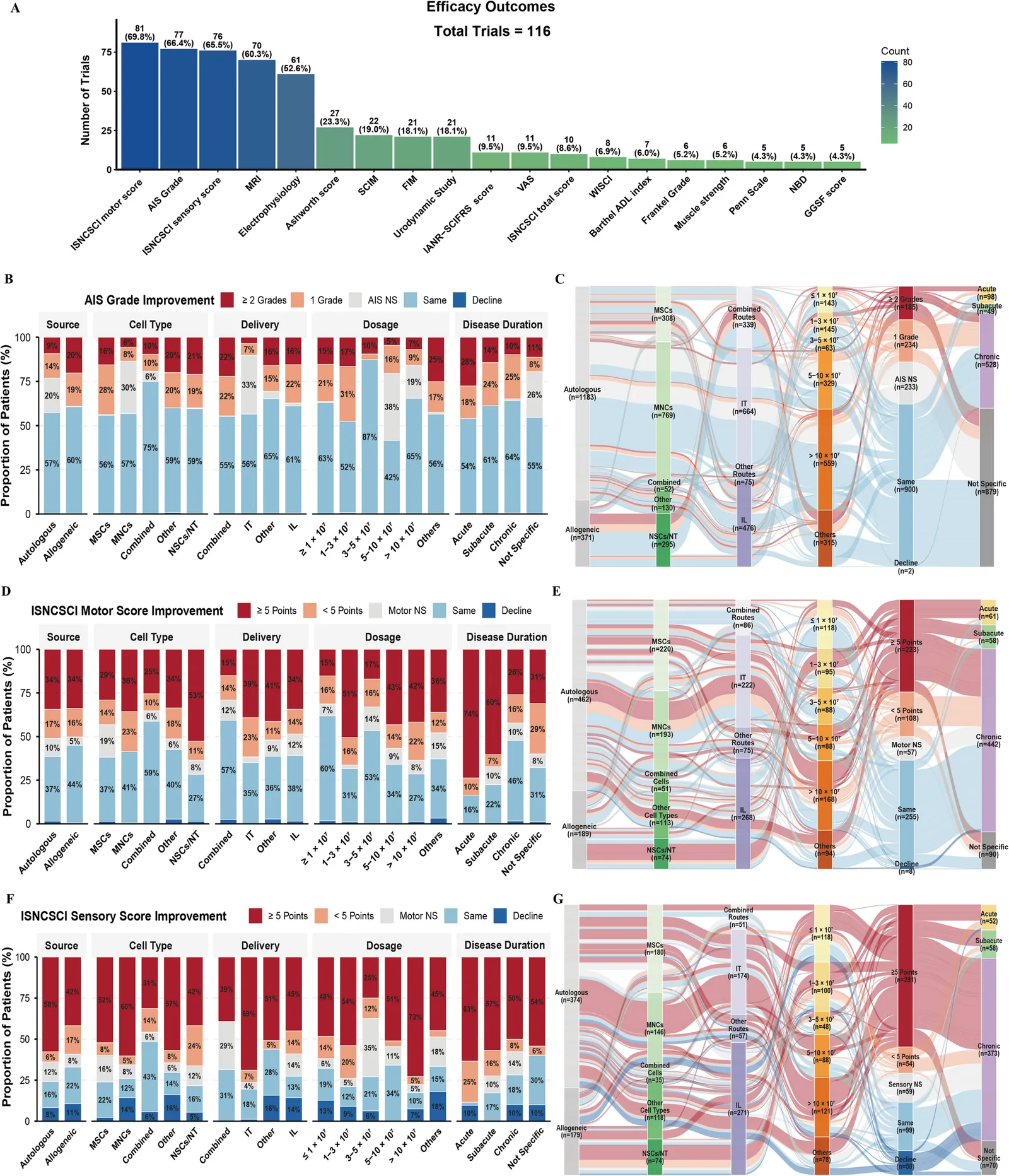
Comprehensive Landscape of Efficacy Outcomes and Patient-Level Neurological Recovery Profiles Stratified by Therapeutic Parameters and Disease Phases. **A** Frequency distribution of clinical efficacy metrics utilized across the 116 included trials. Only outcome measures reported in ≥ 5 trials are displayed, with the corresponding absolute number of trials and percentages annotated above each bar. **B**, **C** Stratified relative proportions (%, B) and comprehensive multi-column alluvial diagrams (**C**) detailing patient-level trajectories for transitions in the American Spinal Injury Association Impairment Scale (AIS) grade. Data are systematically cross-stratified by key clinical parameters, including cell source, cell type, delivery route, and administration dosage, and notably extended to examine the impact of clinical timing (Study Phase: Acute, Subacute, Chronic, and Not Specific). Neurological outcomes are color-coded based on severity and magnitude of change: improvement of ≥ 2 grades (deep red), 1 grade (light orange), non-specified improvement (AIS NS, grey), no change (Same, light blue), and neurological decline (deep blue). In B, proportions < 4% are omitted from data labeling to ensure visual clarity. In C, each vertical column represents an independent clinical covariate layer (from left to right: Source to Study Phase), where the height of each block (stratum) reflects the absolute patient pool size (n) within that sub-category. Colored alluvial streams tracking from left to right illustrate the proportional flow of specific neurological outcomes across successive therapeutic dimensions, mapping the multi-variable correlation between initial cell therapy parameters and final clinical timing. (**D**, **E**) Proportional (**D**) and absolute patient-flow trajectory distributions (**E**) of ISNCSCI motor score changes across distinct therapeutic parameters and disease phases. Therapeutic efficacy gains are categorized by quantitative thresholds into ≥ 5 points, < 5 points, motor non-specified (Motor NS), Same, and Decline. The downstream alluvial splitting and convergence toward the final Study Phase layer in E visually demonstrate how distinct operational parameters interlock with clinical intervention windows to shape motor score recovery. (**F**, **G**) Proportional (**F**) and absolute patient-flow trajectory distributions (**G**) of ISNCSCI sensory score changes across therapeutic parameters and disease phases, evaluated under identical stratification and visualization rules as the motor score frameworks. Abbreviations: ASIA, American Spinal Injury Association; AIS, American Spinal Injury Association Impairment Scale; ISNCSCI, International Standards for Neurological Classification of Spinal Cord Injury; MRI, Magnetic Resonance Imaging; SCIM, Spinal Cord Independence Measure; FIM, Functional Independence Measure; IANR-SCIFRS, International Association of Neurorestoratology Spinal Cord Injury Functional Rating Scale; VAS, Visual Analog Scale; WISCI, Walking Index for Spinal Cord Injury; ADL, Activities of Daily Living; NBD, Neurogenic Bowel Dysfunction; GGSF, Goal Attainment Scaling; MSCs, Mesenchymal Stromal Cells; MNCs, Mononuclear Cells; NSCs/NT, Neural Stem Cells / Neural Tissue; IT, Intrathecal; IL, Intralesional; NS, Not Specified

To systematically evaluate the therapeutic efficacy of cellular interventions, we first mapped the landscape of clinical outcome measures utilized across the 116 included trials (Fig. 5A). The American Spinal Injury Association (ASIA) impairment scale, which is based on the standardized neurological examination according to the International Standards for Neurological Classification of Spinal Cord Injury (ISNCSCI) and its associated motor and sensory scoring systems, emerged as the predominant assessment tools, employed in the majority of studies. Beyond the neurological assessment according to ISNCSCI, magnetic resonance imaging (MRI) was the most frequently utilized objective radiological metric. Functional independence and secondary complication metrics, such as the Spinal Cord Independence Measure (SCIM) and the Visual Analog Scale (VAS) for pain assessment, were utilized less frequently, appearing in 22 trials (19.0%) and 11 trials (9.5%) respectively.

Patient-level analysis of AIS grade transitions revealed a broadly stable neurological profile following cell therapy interventions, although recovery trajectories were significantly influenced by the timing of the intervention (Fig. 5B, **C**). A substantial proportion of patients exhibited no shift in AIS classification, remaining at their baseline grade. For example, stability was observed in 57% of patients receiving autologous cells and 60% of those receiving allogeneic treatments. To address the confounding effect of spontaneous neurological recovery, we explicitly stratified these outcomes by disease duration. In the acute phase cohort, 28% of patients achieved an improvement of two or more grades. However, without concurrent control arms in the acute and subacute phases, it is nearly impossible to distinguish between natural spontaneous recovery and actual treatment effects. In contrast, among patients treated in the chronic phase (total *n*= 528), where spontaneous neurological improvement is generally minimal, 337 patients (63.8%) remained stable, while 132 patients (25.0%) achieved an improvement of one AIS grade and 52 patients (9.8%) demonstrated an improvement of two or more AIS grades. An additional 5 patients (0.9%) were reported to have improved in AIS classification, although the magnitude of the grade change was not specified. These specific neurological gains observed in the chronic cohort are more reliably attributable to the cellular therapy itself. Across specific cellular modalities, among the subset of patients who achieved an improvement in their AIS classification, a single-grade transition was the most frequent outcome, noted in 28% of individuals treated with MSCs and 30% of those receiving MNCs. Improvements of two or more grades were observed in a smaller subset, representing 20% of the allogeneic group and 16% of the MSC group [59–62]. Importantly, overt neurological decline based on overall AIS grading was documented in only 2 patients within the chronic cohort.

Further detailed analysis focused on quantitative changes in ISNCSCI motor scores (Fig. 5D, **E**). The total motor score represents the comprehensive sum of upper and lower extremity motor scores, with a maximum score of 100 points. Recovery profiles were categorized by quantitative threshold gains, specifically improvements of 5 motor score points or greater, improvements of less than 5 motor score points, and no change or decline. To account for the confounding influence of natural disease progression, we stratified these outcomes by injury chronicity. In the acute and subacute cohorts, 74% and 60% of patients achieved an improvement of 5 motor score points or greater, respectively. However, without concurrent control arms in the acute and subacute phases, it is nearly impossible to distinguish between natural spontaneous recovery and actual treatment effects. In contrast, within the chronic phase cohort where spontaneous motor recovery is clinically plateaued, 115 out of 442 patients (26.0%) achieved an absolute improvement of 5 motor score points or greater. Among the 37 analyzed studies involving chronic patients that utilized ISNCSCI motor scores, 13 studies reported patients achieving this threshold, with an average motor score improvement of 3.80 points across the chronic cohort. This specific neurological improvement in the chronic population provides a more reliable indicator of the biological efficacy of the cellular treatments. Stratification by specific cellular modalities revealed distinct recovery distributions: an improvement of 5 motor score points or greater was observed in 53% of patients treated with NSCs/NT, 36% of those receiving MNCs, and 29% of individuals administered MSCs. Conversely, distinct motor function decline was recorded in 8 out of the 651 total analyzed patients. These instances of neurological deterioration were reported across 5 independent studies, affecting 7 patients in the chronic phase and 1 patient with an unspecified injury phase [63–67]. This functional deterioration represents a safety concern that warrants further evaluation within the context of clinical trial design.

Finally, we evaluated the corresponding changes in ISNCSCI sensory scores using an identical quantitative threshold categorization (Fig. 5F, **G**). The ISNCSCI sensory score represents the comprehensive sum of both light touch and pinprick assessments, with a maximum total score of 224 points. Sensory score trajectories exhibited greater variability and divergent trends compared to motor outcomes. In the acute and subacute cohorts, 63% and 57% of patients, respectively, achieved a total sensory score improvement of 5 points or greater. However, consistent with our evaluation of motor outcomes, without concurrent control arms in the acute and subacute phases, it is nearly impossible to distinguish between natural spontaneous recovery and actual treatment effects. Within the chronic phase cohort, where spontaneous sensory gains are limited, 187 out of 373 patients (50.1%) achieved an improvement of 5 sensory score points or greater. Stratification by specific cellular modalities revealed that an improvement of 5 sensory score points or greater was observed in 60% of patients receiving MNCs, 52% of individuals administered MSCs, and 42% of those treated with NSCs/NT. Neurological decline in sensory function, strictly defined as an absolute decrease in the total combined sensory score, was documented in 50 patients overall. When stratified by administration route, sensory decline was observed in 14% of patients receiving intralesional injections, whereas it was rarely recorded in the intrathecal administration cohort. This disparity aligns with the inherent procedural risks of direct intramedullary transplantation, which carries a higher probability of mechanically disrupting preserved neural pathways compared to cerebrospinal fluid delivery. Therefore, interpreting these functional impairments requires careful consideration of the specific delivery modality and its associated surgical risks.

### Characterization of clinical trial phases and designs

**Fig. 6:**
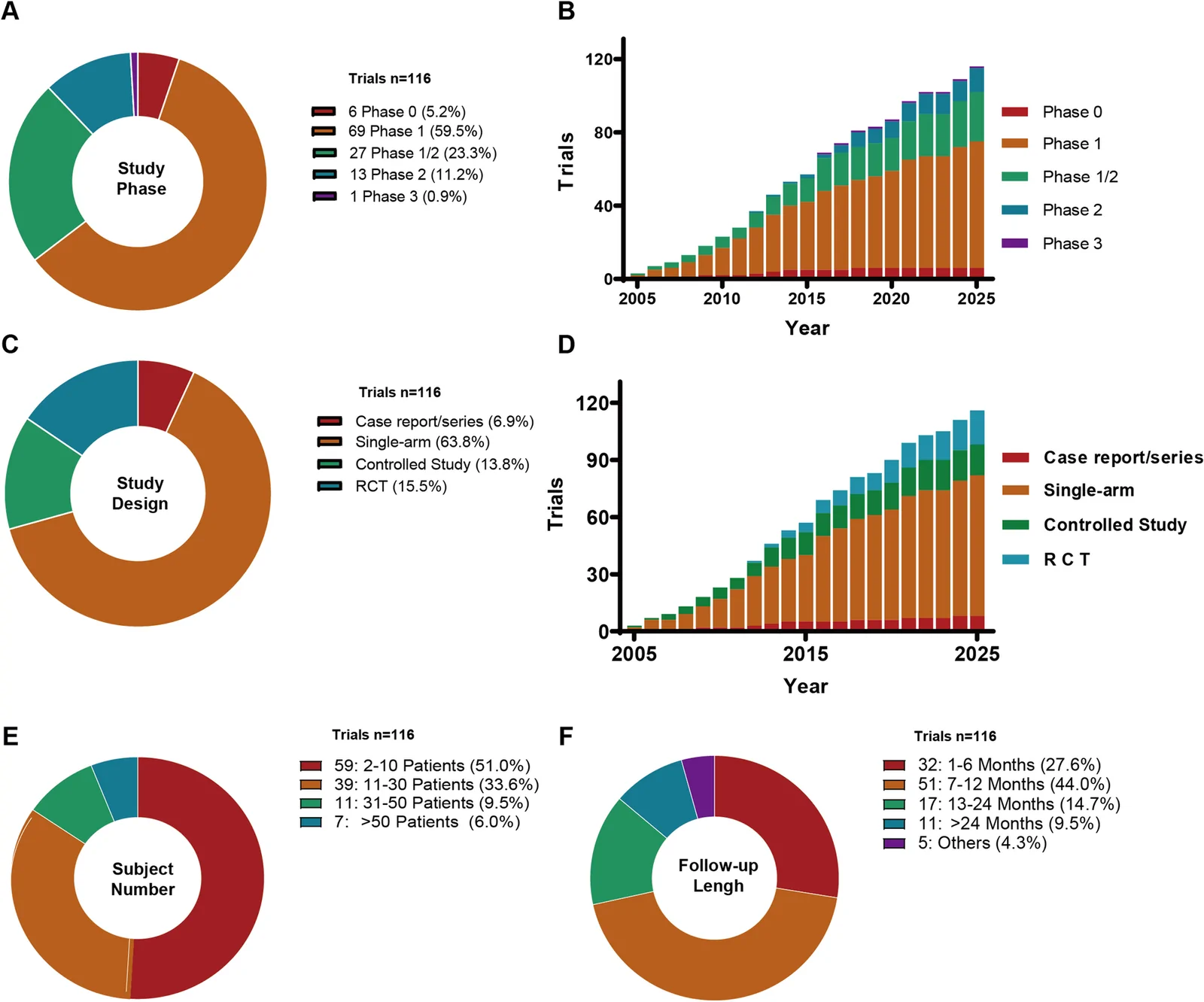
Landscape of Study Design and Methodological Characteristics in Cell Therapy for Spinal Cord Injury. **A** Distribution of clinical trial phases. **B** Temporal trends in study phases from 2005 to 2025. **C** Distribution of clinical trial designs. **D** Temporal trends in study designs from 2005 to 2025. **E** Distribution of subject enrollment numbers per trial. **F** Distribution of follow-up durations. Abbreviations: RCT: randomized controlled trial

Our analysis of the 116 identified clinical trials reveals that the current landscape of cell therapy for SCI is predominantly characterized by early-phase investigations (Fig. 6A). Specifically, Phase 1 trials constitute the majority, accounting for 59.5% (69/116) of the studies included. In contrast, trials that have progressed to later stages are markedly scarce; Phase 2 trials represent only 11.2% (13/116), and Phase 3 trials are exceptionally rare, comprising a mere 0.9% (1/116). Notably, the sole Phase 3 study identified was published in 2015, and no subsequent Phase 3 trials have been reported in the ensuing years (Fig. 6B), suggesting a potential bottleneck in the translation to pivotal clinical stages.

Consistent with this distribution of study phases, the experimental designs employed in these trials largely reflect exploratory methodologies (Fig. 6C). A significant proportion of the research lacks rigorous control groups, as single-arm studies (63.8%) and case reports or series (6.9%) collectively account for over 70% of the total dataset. Conversely, high-evidence designs remain limited, with randomized controlled trials (RCTs) representing only 15.5% of the analyzed studies. This trend has persisted over time (Fig. 6D), indicating that the field has not yet fully transitioned toward large-scale, comparative efficacy verifications.

Furthermore, the scale of participant enrollment underscores the preliminary nature of these investigations (Fig. 6E). We observed that more than half of the studies (51.0%) enrolled a modest cohort of 2 to 10 patients. Larger clinical trials are infrequent, with only seven studies (6.0%) involving more than 50 participants. Regarding the duration of post-treatment monitoring, the follow-up periods are predominantly short-term (Fig. 6F). The cumulative data show that over 70% of the studies implemented a follow-up duration of one year or less (27.6% for 1–6 months and 44.0% for 7–12 months). Studies providing long-term data exceeding two years are a minority, accounting for just 9.5% of the total.

Collectively, these findings demonstrate that the clinical application of cell therapy for SCI remains in a nascent developmental stage. Given the prevalence of early-phase, single-arm, and small-sample studies with limited follow-up, drawing definitive conclusions regarding therapeutic efficacy is currently challenging. Consequently, the primary focus of the existing body of literature remains centered on evaluating safety and feasibility rather than confirming robust clinical benefits.

## Discussion

### Patient selection: demographic representation and strategic mismatches

A primary impediment to bridging the translational gap remains the lack of precision in patient stratification. Current inclusion criteria rely predominantly on macroscopic demographic variables, such as injury level and AIS grade. These metrics fail to account for the complex microscopic heterogeneity of SCI pathology [68]. This lack of stratification stands in sharp contrast to emerging standards in pharmacological interventions. Trials investigating Riluzole, Minocycline, and Anti-Nogo-A antibodies have successfully pioneered the use of specific biomarkers to stratify patient cohorts and objectively monitor biological responses [69, 70]. Conversely, the cell therapy landscape remains reliant on coarse clinical metrics. Although sporadic reports suggest that biomarkers such as GFAP may fluctuate following MSC therapy, the field lacks a systematic integration of imaging, fluid, and electrophysiological biomarkers to identify the specific sub-populations most likely to benefit [70–73]. Furthermore, prevailing trial designs exhibit a strategic mismatch between clinical necessities and experimental pragmatism.

Regarding injury levels, thoracic SCI were targeted in 25 trials, while cervical SCI was specified in 17 trials. The available data indicate that interventions in thoracic SCI patients constitute a substantial proportion of the documented clinical landscape. This distribution highlights the current focus of cell therapy trials among the subset of studies providing respective information. However, because the overall landscape leans toward thoracic SCI, clinical evidence specifically addressing the restoration of upper extremity and hand function, which remains the paramount priority for patients with cervical SCI, remains comparatively limited. Similarly, a temporal mismatch exists where trials predominantly recruit chronic patients. While this timing circumvents the acute neuroinflammatory response, the primary drivers for this specific patient selection strategy are rooted in clinical safety and trial methodology. Specifically, enrolling chronic patients helps avoid acute phase complications (such as severe infections and surgery associated instability). Most importantly, it provides a stable neurological baseline. Particularly in low sample size single arm trials, a stable chronic baseline is essential for investigators to differentiate true intervention driven improvements from the spontaneous natural recovery that typically confounds acute trials. This cautious enrollment strategy occurs despite the acute and subacute phases offering a critical temporal window to effectively modulate the early pathological cascade and facilitate active neural regeneration [74, 75].

Furthermore, our demographic analysis highlights an evolving trend regarding patient age that warrants consideration. While the study cohorts are predominantly composed of young to middle-aged adults, there is a gradual upward trajectory in the average age of enrolled patients, rising from approximately 30.3 years in 2005 to 36.4 years in recent trials. This demographic shift within clinical trials likely mirrors the changing real-world epidemiology of SCI, which is increasingly influenced by the global increase in life expectancy and a growing proportion of fall-related injuries among older populations [76]. However, because standard clinical trial designs typically employ strict upper age limits (frequently 60 to 65 years) to minimize confounding variables introduced by age-related comorbidities and ensure procedural safety, the mean age of trial participants inherently remains anchored in the younger to middle-aged bracket [77]. Consequently, while current clinical trials are gradually reflecting the global aging tendency, the derived safety and efficacy data are still inherently restricted to non-geriatric populations.

Additionally, the predominant enrollment of patients with sensorimotor complete SCI (AIS grade A), particularly in early stage trials, is primarily driven by strict clinical safety and ethical considerations. Because invasive cellular administration carries an inherent risk of inflicting iatrogenic damage to preserved neural tissue, patients with incomplete SCI are frequently excluded to prevent any further neurological compromise. Consequently, the fundamental priority remains the avoidance of procedural harm to intact spinal cord structures [22]. Beyond this ethical requirement, patient selection should carefully navigate the confounding effects of spontaneous recovery. It is important to recognize that patients with sensorimotor complete SCI can also exhibit some degree of natural recovery, particularly in the acute and subacute phases. However, beyond the first year post-injury, spontaneous neurological recovery is almost completely absent. This critical stabilization applies to both complete and incomplete injuries in the chronic phase. Therefore, recruiting chronic patients provides a highly stable baseline, enabling researchers to reliably attribute any observed sensorimotor improvements to the cellular intervention rather than natural disease progression. While incomplete injuries present significant statistical noise and ceiling effects during the early phases of injury, these challenges are largely mitigated in the chronic setting. Nevertheless, because current efficacy data are derived primarily from strictly selected cohorts with complete injuries, these findings may ultimately lack broad generalizability across the entire clinical spectrum of SCI.

The current reliance on clinical variables for patient selection, such as neurological injury level and baseline injury severity, inherently limits precision. These conventional clinical metrics often fail to account for the profound heterogeneity of spinal cord pathology [68]. While our research confirms that broad clinical grading remains the absolute standard inclusion criterion across current cellular therapies, future trial designs might eventually benefit from the advanced stratification strategies emerging in pharmacological interventions [69, 70]. Moving forward, the gradual integration of specific fluid biomarkers, advanced imaging, and neurophysiological metrics capable of detecting sub-threshold or latent neural connectivity could help identify specific patient sub-populations most likely to respond to cellular interventions, thereby refining future cohort selection and bridging the current translational gap [71–73].

### Intervention characteristics: the dosing paradox and cell-lineage specificity

Regarding the intervention itself, our analysis revealed a seemingly paradoxical inverse correlation in which lower cell dosages were associated with higher frequencies of specific adverse events. This finding should not be interpreted as dose-dependent toxicity; rather, it represents a confounding effect driven by cell-type specificity. High-dose protocols are predominantly utilized in trials involving MSCs, which possess low immunogenicity and a robust safety profile that permits extensive expansion [78,79]. In contrast, low dose regimens are typically mandated for cellular lineages categorized as high risk due to their documented potential for tumorigenesis, severe immunogenic reactions, or ectopic differentiation [80]. Historical clinical evidence from specific case reports and small series has directly implicated certain lineages, notably olfactory ensheathing cells and macrophages, in severe adverse events including intramedullary tumor formation, pulmonary embolism, and osteomyelitis [81–84]. Following the publication of these profound safety warnings, our longitudinal analysis reveals a complete and abrupt global cessation of clinical trials utilizing these specific cellular modalities after 2015. This halt in related publications provides circumstantial evidence that the scientific community actively recalibrated the therapeutic profile of these interventions, practically concluding that their inherent biological risks effectively outweigh their clinical utility. Therefore, the observed adverse events in low dose groups reflect the intrinsic biological risks of the cell source rather than the dosage itself. Consequently, future dosing guidelines must be stratified by cell lineage rather than applied universally.

### Synthesizing efficacy determinants and neurological recovery profiles

Our synthesis reveals that while overall neurological stability is the most common outcome following cellular therapy for spinal cord injury, the interpretation of therapeutic efficacy is fundamentally contingent upon the chronological phase of the injury. We acknowledge that in acute and subacute cohorts, assigning causality to the cellular intervention is methodologically challenging. As observed improvements cannot be easily distinguished from spontaneous natural recovery in the absence of concurrent or historical control groups, acute and subacute efficacy data must be interpreted with extreme caution. Consequently, our evaluation of actual biological efficacy is anchored predominantly on the chronic cohort, where spontaneous recovery has clinically plateaued. By systematically comparing varying cellular interventions within this context, distinct patterns of efficacy emerge across different functional domains.

Regarding cellular source, autologous and allogeneic therapies demonstrated comparable efficacy in motor recovery. Both sources facilitated a 5 point or greater motor gain in approximately one third of the treated patients and maintained high rates of overall American Spinal Injury Association Impairment Scale stability. However, sensory outcomes exhibited notable divergence. Autologous sources yielded a higher proportion of robust sensory improvements but paradoxically presented a slightly higher incidence of distinct sensory decline. This discrepancy suggests that while allogeneic products might offer highly standardized microenvironmental modulation, autologous cells could elicit more potent but potentially heterogeneous local tissue responses depending on individual patient biology.

Analysis of specific cell lineages highlighted distinct differentiated recovery profiles. MSCs and mononuclear cells provided consistent and steady improvements, dominating the single step scale conversions. In contrast, interventions utilizing NSCs/NTs were associated with a pronounced capacity for motor recovery, with over half of that specific cohort achieving significant motor gains. Yet, this aggressive motor recovery was accompanied by the highest absolute rate of sensory decline among all cell types evaluated. This dichotomy may reflect fundamental biological and procedural differences between cellular therapies. MSCs primarily exert broad neuroprotective and immunomodulatory paracrine effects. Conversely, interventions utilizing NSCs frequently involve direct intralesional delivery and rely on cells with substantial proliferative capacity. Therefore, the observed sensory decline may be largely attributable to mechanical factors rather than aberrant synaptic integration. Specifically, the invasive nature of direct intralesional transplantation inherently carries the risk of inflicting iatrogenic damage upon preserved sensory pathways. Furthermore, subsequent in vivo graft expansion could secondarily compress adjacent eloquent axonal tracts, thereby precipitating localized sensory deterioration.

The route of administration also critically influenced the balance of therapeutic outcomes. While intrathecal and intralesional deliveries yielded comparable overall rates of motor score improvement, these comparative observations must be interpreted cautiously, as delivery route preferences often co vary with the injury phase, potentially confounding outcomes with spontaneous natural recovery. Nevertheless, intrathecal administration demonstrated a clear advantage in driving robust sensory gains while maintaining a lower profile of neurological decline. This divergence likely reflects the distinct mechanistic profiles of these delivery methods. Intralesional treatment works by directly depositing cellular grafts into the epicenter of the injury to physically bridge the tissue defect and highly concentrate local trophic factors. However, this direct intraparenchymal injection is highly invasive and carries the inherent risk of mechanically disrupting adjacent preserved eloquent tracts, which may precipitate sensory deterioration. In contrast, intrathecal treatment relies on the cerebrospinal fluid circulation to broadly distribute cells and their secreted paracrine factors across the spinal axis. Because the majority of patients in the analyzed trials were treated in the subacute or chronic phases, the underlying therapeutic mechanism is less likely to reflect early neuroprotection. Rather, the extensive biodistribution afforded by intrathecal delivery likely facilitates widespread trophic support, sustained immunomodulation, and the promotion of neuroplasticity along the preserved sensory pathways, optimizing tissue repair without the localized iatrogenic trauma inherently associated with direct parenchymal penetration.

Finally, the evaluation of cell dosage identified a highly complex landscape, emphasizing that direct correlations between absolute cell numbers and therapeutic effects must be drawn with extreme caution. Our initial observation of varying recovery rates across different dosage tiers does not imply a universal optimal therapeutic window. Rather, it highlights the impossibility of establishing a standardized numerical dosage across heterogeneous cellular interventions. Different cell lineages possess distinct physical dimensions, creating distinct volumetric footprints when grafted into a confined anatomical space such as the spinal cord. Furthermore, these therapies utilize fundamentally different modes of action. Cells intended to serve as physical scaffolds for axonal regeneration inherently possess completely different quantitative prerequisites compared to those utilized primarily as sources of secreted molecular factors to modulate the injury microenvironment. Therefore, while our data indicate that extreme cellular concentrations can result in highly heterogeneous sensory responses and potential functional decline, future clinical protocols must avoid generalized numerical thresholds. Optimal dosages must be rigorously tailored to the specific physical size, biological purpose, and delivery route of the utilized cell lineage.

### Comparators and confounders: the methodological black box

The valid interpretation of efficacy and safety data in allogeneic transplantation is critically dependent on the management of concurrent therapies, most notably immunosuppressive regimens. As established in our systematic results, the application of these drugs exhibits a distinct lineage-dependent pattern, utilized predominantly alongside NSCs but omitted with MSCs. While our data indicate that these protocols are generally tolerated, it is important to recognize the baseline infectious risks inherent to this patient population. Patients with SCI possess a well documented susceptibility to infections, particularly urinary tract infections that can progress to sepsis. The concurrent administration of systemic immunosuppressive regimens may further exacerbate this underlying vulnerability, thereby increasing the overall risk of severe infectious complications [85]. However, despite extracting these available data, the precise evaluation of therapeutic efficacy remains significantly compromised by persistently opaque reporting. This creates a methodological black box that obscures the true therapeutic effect of the cellular graft. Even among the trials that explicitly employed potent agents such as tacrolimus or mycophenolate mofetil to mitigate host versus graft rejection, crucial protocol details including specific dosages, treatment durations, and tapering schedules are frequently absent from published methodologies. In several instances, studies acknowledge the use of immunosuppression generically without specifying the pharmacological agent.

The lack of standardization in immunosuppressive protocols introduces methodological challenges into both safety and efficacy analyses. From a safety perspective, inadequate immunosuppression may expose patients to immunogenic reactions. Conversely, the administration of these pharmacological agents can complicate the clinical picture by interacting with existing baseline morbidities. It is well recognized that infections and gastrointestinal dysfunctions are prevalent complications inherent to the natural course of SCI. The addition of systemic immunosuppressive regimens likely exacerbates these underlying vulnerabilities, thereby amplifying the patient’s susceptibility to such adverse events, as reflected in our extracted data. Furthermore, preclinical evidence suggests that certain immunosuppressants may directly modulate the viability and differentiation potential of the transplanted cells, thereby independently altering the biological efficacy of the graft. To definitively isolate the therapeutic contribution of cell transplantation, future study designs must mandate rigorous, transparent, and highly standardized reporting of all concomitant immunosuppressive protocols [86].

Similarly, physical rehabilitation represents a potent yet poorly controlled variable. Its confounding impact should be carefully contextualized within the phase of SCI. It is essential to differentiate between neurological and clinical functional outcomes. Typical rehabilitation interventions do not impact neurological improvements in either the acute or chronic phases. However, these interventions strongly influence functional recovery in the acute and subacute settings. Furthermore, in chronic patient cohorts, rehabilitation may still improve functional measures and activities of daily living, although to a very limited extent. Therefore, it remains a variable that requires analytical attention. In acute and subacute trials, active rehabilitation is both highly effective and ethically mandated as the standard of care. Because it is clinically unfeasible to omit rehabilitation, this concurrent therapy must be carefully managed. Currently, researchers rarely quantify the frequency, intensity, or specific modalities employed, introducing statistical noise. Recognizing that imposing a rigid and uniform rehabilitation protocol across multicenter trials is clinically unrealistic, future study designs should instead focus on comprehensive tracking. We recommend the standardized monitoring and detailed quantification of physical rehabilitation interventions evenly across all experimental and control cohorts, allowing for appropriate statistical adjustments without compromising patient care [87].

### Outcome assessment: refining safety and efficacy metrics

In the evaluation of safety outcomes, the evolution of reporting suggests a potential utility in distinguishing between directly cell transplant related complications and those resulting from procedural interventions. Events such as fever likely associated with MSC transplantation, may reflect acute cytokine release and immunomodulatory activities. In contrast, procedural events, such as positional headache following intrathecal injection, are generally consequences of the puncture of the intrathecal space and the resulting transient intracranial hypotension. Although these events are often transient, the target patient population already experiences complications inherently associated with the condition. Therefore, the proactive management of these procedural side effects warrants careful consideration to minimize additional clinical burden [88].

Beyond these transient events, the potential for procedure-associated sensorimotor impairment represents a more severe clinical concern. As observed in our quantitative synthesis, neurological decline involves multifactorial etiologies. Regarding sensory deterioration, interventions necessitating direct intramedullary transplantation inherently carry a procedural risk of mechanically disrupting preserved intact neural pathways compared to cerebrospinal fluid delivery. Conversely, reports of motor decline highlight both procedural and pathological factors. Documented procedural causes include severe complications, such as surgical instrumentation-induced aseptic meningitis leading to subsequent spinal cord edema [66]. Additionally, motor decline observed in control cohorts within cellular therapy trials suggests that the natural progression of SCI, including ongoing Wallerian degeneration, may independently contribute to late neurological loss [65]. Consequently, navigating these safety concerns requires procedural infection control, the careful consideration of delivery modalities and their associated surgical risks, and a clear differentiation between iatrogenic complications and natural disease progression.

Transitioning to the evaluation of therapeutic efficacy, our systematic analysis of clinical metrics indicates a concentrated reliance on specific evaluation tools across current trial designs. Metrics derived from the standardized neurological assessment according to ISNCSCI serve as the primary assessment tools. However, given the nuanced biological activity of cellular therapies, supplementing this framework with additional modalities such as electrophysiological assessments offers a more granular and objective perspective. The continued application of these measures is beneficial because they provide objective quantification of spinal cord conduction across the lesion site, independent of subjective clinical grading. By identifying functionally spared neuronal pathways, these assessments capture residual connectivity that standard clinical scales may overlook. For example, recent data from a phase 2b trial evaluating the plasticity-inducing antibody NG101 demonstrated that electrophysiological stratification using somatosensory evoked potentials successfully identified a responsive patient subgroup, thereby increasing the observed treatment effect sizes and reducing the required sample sizes [89]. Furthermore, combining these electrophysiological measures with structural imaging biomarkers substantially enhanced the detection of clinical treatment effects [90]. Consequently, the integration of available tools, including ISNCSCI, electrophysiology, and imaging, primarily serves to measure tissue sparing. This enables a better characterization of patients who are more likely to respond to interventions. However, it is important to acknowledge that this stratification approach comes at the cost of testing interventions within a highly selected population [91].

Furthermore, our quantitative mapping shows that multidimensional functional assessments are less frequently employed. Metrics evaluating functional independence, notably the SCIM, were utilized in a smaller subset of the analyzed studies (less than 20%)^92^. Expanding the evaluation matrix beyond traditional sensorimotor indices to encompass these targeted functional domains provides a broader assessment of patient recovery [93–95]. Therefore, incorporating a multidimensional approach in future trial designs may facilitate the capture of a wider spectrum of treatment outcomes [96, 97].

#### Study design, the phase II dilemma, and future clinical trajectories

Viewing the landscape through the lens of study design, the trajectory of cellular interventions over the past two decades signifies a successful transition from preclinical proof of concept to the validation of primary feasibility [98]. Clinical data confirm that transplantation is feasible in humans. However, the field currently faces a translational bottleneck, often characterized as a Phase II dilemma. The limited number of advanced-phase RCTs restricts the comprehensive evaluation of concurrent efficacy and safety. Furthermore, the reliability of early phase efficacy data warrants cautious interpretation, as many preliminary investigations deviate from rigorous design standards. Consequently, while foundational safety is established, the definitive validation of clinical efficacy requires the systematic adoption of more rigorous methodological and protocol standards in future trial designs.

To determine the true efficacy of defined cellular grafts and overcome this dilemma, future clinical investigations must adopt rigorous methodological frameworks. An analysis of the registered clinical trial pipeline from 2021 to 2025 provides a concrete outlook on how these advanced trials should be structured **(**Table 1**)**. First, there must be a transition away from exploratory single arm protocols toward controlled trial designs. We acknowledge that executing strict double blind RCTs for invasive interventions presents significant ethical barriers, particularly regarding the use of saline as a placebo [88]. For patients in the chronic phase, the implementation of crossover designs represent one potential approach that maintains statistical validity by providing internal controls while allowing all participants to eventually receive the therapeutic intervention [99]. However, this approach is not feasible in the acute or subacute phases due to the confounding effects of spontaneous recovery. In these earlier phases, an alternative strategy is to enrich the treated cohort using matched historical or synthetic controls. Recent studies have demonstrated that utilizing data-driven recovery predictions to generate synthetic controls provides a viable alternative to randomization, reducing heterogeneity in acute and subacute SCI trials [100, 101].

**Table 1:**
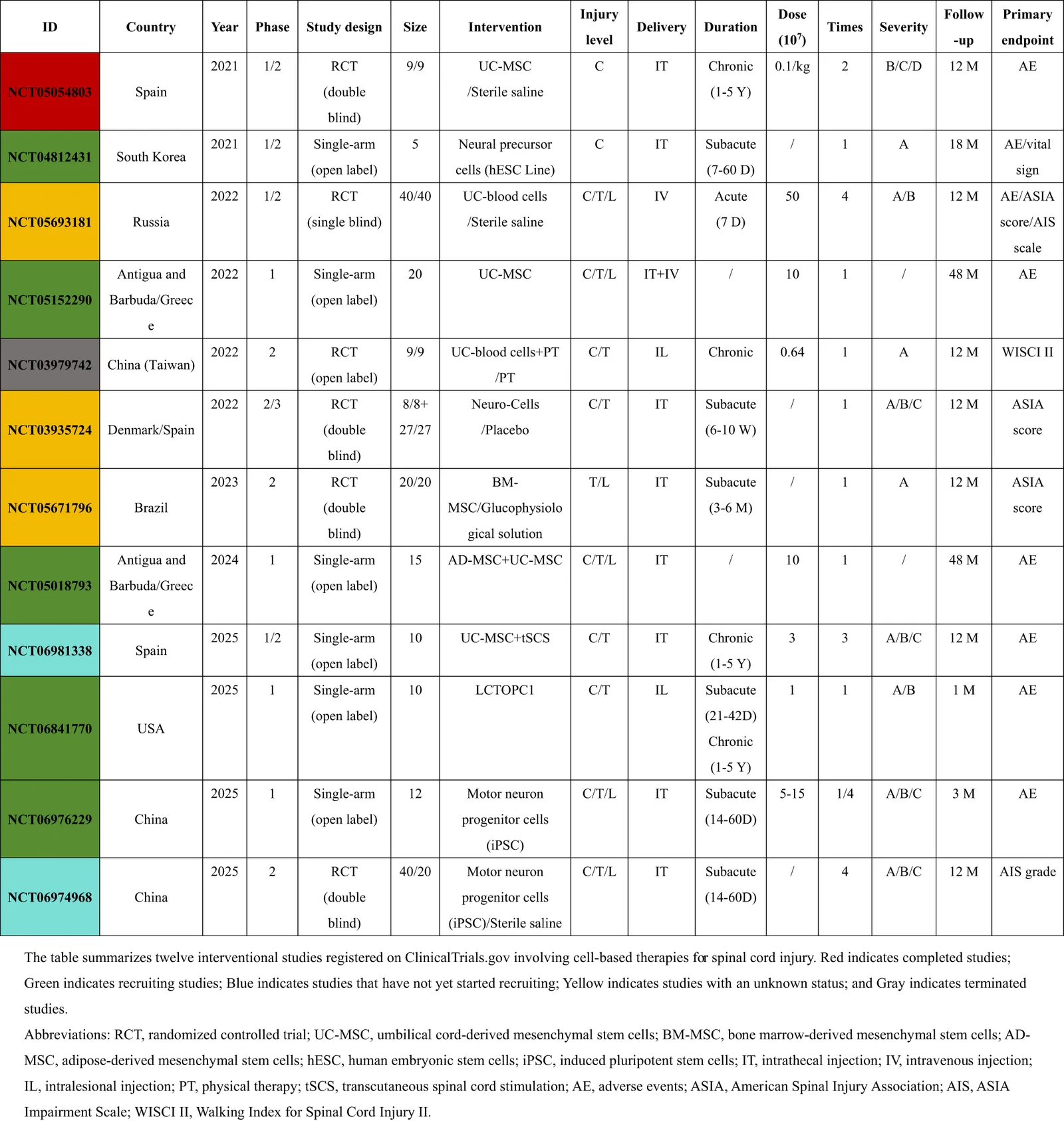
Characteristics of Registered Clinical Trials for Cell Therapy in Spinal Cord Injury (2021–2025)

Furthermore, the careful refinement of execution protocols is necessary to better isolate therapeutic variables. Based on our extracted data, intrathecal injection demonstrates certain advantages, particularly regarding its less invasive profile compared to direct intraparenchymal approaches. However, the appropriate delivery route cannot be generalized; rather, it should be specifically determined by the biological characteristics of the chosen cell type and its envisioned mechanism of action. Additionally, exploring appropriate administration windows and dosing strategies remains an area for future investigation. While some recent trials are exploring repeated dosing schedules to potentially prolong the therapeutic window, we acknowledge that making definitive recommendations based on current research is challenging, primarily due to the confounding influence of the disease phase across different studies [102, 103]. Regarding patient monitoring, rather than mandating unfeasibly prolonged observation periods in a single trial, a more pragmatic approach may involve separating shorter-term efficacy assessments from distinct long-term safety follow-up registries [104–107]. Finally, regarding patient monitoring, rather than imposing unfeasibly prolonged observation periods in a single trial, a more viable approach may involve separating shorter-term efficacy assessments from distinct long-term safety follow-up registries. By cautiously refining these trial design parameters, the field can navigate beyond the current translational bottleneck toward more reliable clinical translation [30, 108].

## Supplementary Information

Below is the link to the electronic supplementary material.

Supplementary Material 1: Additional file: Table S1-S5, Figure S1-S2

## Data Availability

All study data have been archived in this manuscript, supplementary materials, and original trial publications, with analysis codes accessible upon formal request through correspondence with the corresponding author following established academic protocols for data transparency.
