# Supplementary material for: Clinical trial landscape of cell therapy for spinal cord injury: from integrated practices to future developments
Source: BMC Med. 2026 Jul 20;24:486. doi: 10.1186/s12916-026-05078-2 (PMC13579809; doi:10.1186/s12916-026-05078-2)
Supplement: Supplementary file 1 [file 12916_2026_5078_MOESM1_ESM.docx]

**Clinical Trial Landscape of Cell Therapy for Spinal Cord Injury: From Integrated Practices to Future Developments**

**Supplementary Material**

**Table of Content**

1. Supplementary Tables	2
2. Supplementary Figures	22

**Web of science**

((TS= (“Spinal cord injury” OR “Spinal injury” OR “Spinal Cord Trauma” OR “Spinal Cord Transection” OR “Spinal Cord Laceration” OR “Post-Traumatic Myelopathy” OR “Spinal Cord Contusion”)) AND TS= (“stem cell” OR “stem cells” OR “mesenchymal stem cell” OR “MSC” OR “macrophage” OR “olfactory ensheathing cells” OR “Schwann cells” OR “neural stem cell” OR “neural progenitor cell” OR “induced pluripotent stem cell” OR “iPSC” OR “oligodendrocyte progenitor cell” OR “OPC”)) AND TS= (clinical OR trials OR trial OR patients OR patient)

**Supplementary Table 1:** Search strategies.

**Supplementary Table 2:** Basic Characteristics of Articles and Trial Design

| **First Author** | **Year** | **Country** | **Journal** | **Title** | **Design** | **Phase** | **Subjects** | **Follow-Up**  **(month)** |
| --- | --- | --- | --- | --- | --- | --- | --- | --- |
| JIANG PC | 2013 | China | EXPERIMENTAL AND THERAPEUTIC MEDICINE | A clinical trial report of autologous bone marrow-derived mesenchymal stem cell transplantation in patients with spinal cord injury | case series | 0 | 20 | 1 |
| CURTIS E | 2018 | USA | CELL STEM CELL | A First-in-Human, Phase I Study of Neural Stem Cell Transplantation for Chronic Spinal Cord Injury | case report | 1 | 4 | 18-27 |
| FESSLER RG | 2022 | USA | JOURNAL OF NEUROSURGERY-SPINE | A phase 1/2a dose-escalation study of oligodendrocyte progenitor cells in individuals with subacute cervical spinal cord injury | single arm | 1/2a | 25 | 12 |
| OH SK | 2016 | South Korea | NEUROSURGERY | A Phase III Clinical Trial Showing Limited Efficacy of Autologous Mesenchymal Stem Cell Therapy for Spinal Cord Injury | single arm | 3 | 16 | 6 |
| IWATSUKI K | 2016 | Japan | NEUROLOGIA MEDICO-CHIRURGICA | A Pilot Clinical Study of Olfactory Mucosa Autograft for Chronic Complete Spinal Cord Injury | single arm | 1/2a | 8 | 24 |
| CHEN L | 2015 | China | CELL TRANSPLANTATION | A Prospective Randomized Double-Blind Clinical Trial Using a Combination of Olfactory Ensheathing Cells and Schwann Cells for the Treatment of Chronic Complete Spinal Cord Injuries | RCT | 2 | 5 | 6 |
| GEFFNER LF | 2008 | Ecuador | CELL TRANSPLANTATION | Administration of Autologous Bone Marrow Stem Cells Into Spinal Cord Injury Patients Via Multiple Routes Is Safe and Improves Their Quality of Life: Comprehensive Case Studies | single arm | 1 | 8 | 24 |
| SHARMA A | 2012 | India | CELL TRANSPLANTATION | Administration of Autologous Bone Marrow-Derived Mononuclear Cells in Children With Incurable Neurological Disorders and Injury Is Safe and Improves Their Quality of Life | single arm | 1 | 4 | 15 |
| SAITO F | 2012 | Japan | RESTORATIVE NEUROLOGY AND NEUROSCIENCE | Administration of cultured autologous bone marrow stromal cells into cerebrospinal fluid in spinal injury patients: A pilot study | single arm | 1 | 5 | 6 |
| VAQUERO J | 2016 | Spain | CYTOTHERAPY | An approach to personalized cell therapy in chronic complete paraplegia: The Puerta de Hierro phase I/II clinical trial | single arm | 1/2a | 12 | 12 |
| ATTAR A | 2011 | Turkey | CYTOTHERAPY | An attempt to treat patients who have injured spinal cords with intralesional implantation of concentrated autologous bone marrow cells | single arm | 1 | 4 | 12 |
| CHERNYKH ER | 2007 | Russia | BULLETIN OF EXPERIMENTAL BIOLOGY AND MEDICINE | Application of autologous bone marrow stem cells in the therapy of spinal cord injury patients | cohort | 1 | 18 | 9 |
| CHHABRA HS | 2016 | India | SPINAL CORD | Autologous bone marrow cell transplantation in acute spinal cord injury-an Indian pilot study | RCT | 1/2a | 14 | 12 |
| KUMAR AA | 2009 | India | EXPERIMENTAL AND CLINICAL TRANSPLANTATION | Autologous Bone Marrow Derived Mononuclear Cell Therapy for Spinal Cord Injury: A Phase I/II Clinical Safety and Primary Efficacy Data | cohort | 1/2a | 297 | 18 |
| SYKOVÁ E | 2006 | Czech Republic | CELL TRANSPLANTATION | Autologous bone marrow transplantation in patients with subacute and chronic spinal cord injury | single arm | 1/2a | 20 | 12 |
| EL-KHEIR WA | 2014 | Egypt | CELL TRANSPLANTATION | Autologous Bone Marrow-Derived Cell Therapy Combined With Physical Therapy Induces Functional Improvement in Chronic Spinal Cord Injury Patients | RCT | 1/2a | 50 | 18 |
| LAMMERTSE DP | 2012 | USA | SPINAL CORD | Autologous incubated macrophage therapy in acute, complete spinal cord injury: results of the phase 2 randomized controlled multicenter trial | RCT | 2 | 33 | 6 |
| BHANOT Y | 2011 | India | BRITISH JOURNAL OF NEUROSURGERY | Autologous mesenchymal stem cells in chronic spinal cord injury | single arm | 1 | 13 | 6 |
| SATTI HS | 2016 | Pakistan | CYTOTHERAPY | Autologous mesenchymal stromal cell transplantation for spinal cord injury: A Phase I pilot study | single arm | 1 | 9 | 12 |
| CHHABRA HS | 2009 | India | SPINAL CORD | Autologous mucosal transplant in chronic spinal cord injury: an Indian Pilot Study | single arm | 1 | 5 | 24 |
| MACKAY-SIM A | 2008 | Australia | BRAIN | Autologous olfactory ensheathing cell transplantation in human paraplegia:: a 3-year clinical trial | cohort | 1/2a | 3 | 36 |
| FÉRON F | 2005 | Australia | BRAIN | Autologous olfactory ensheathing cell transplantation in human spinal cord injury | cohort | 1/2a | 3 | 12 |
| WANG S | 2016 | China | CELL TRANSPLANTATION | Autologous Olfactory Lamina Propria Transplantation for Chronic Spinal Cord Injury: Three-Year Follow-Up Outcomes From a Prospective Double-Blinded Clinical Trial | RCT | 1/2a | 8 | 36 |
| SONG H | 2020 | China | JOURNAL OF HARD TISSUE BIOLOGY | Bone Marrow Mesenchymal Stem Cells Transplantation on Acute Spinal Cord Injury | RCT | 1/2a | 18 | 12 |
| SUZUKI Y | 2014 | Japan | RESTORATIVE NEUROLOGY AND NEUROSCIENCE | Bone marrow-derived mononuclear cell transplantation in spinal cord injury patients by lumbar puncture | single arm | 1 | 10 | 6 |
| AMR SM | 2014 | Egypt | JOURNAL OF SPINAL CORD MEDICINE | Bridging defects in chronic spinal cord injury using peripheral nerve grafts combined with a chitosan- laminin scaffold and enhancing regeneration through them by co-transplantation with bone-marrow-derived mesenchymal stem cells: Case series of 14 patients | case series | 0 | 14 | 24 |
| KISHK NA | 2010 | Egypt | NEUROREHABILITATION AND NEURAL REPAIR | Case Control Series of Intrathecal Autologous Bone Marrow Mesenchymal Stem Cell Therapy for Chronic Spinal Cord Injury | case control | 1/2a | 43 | 12 |
| MOVIGLIA GA | 2009 | Argentina | SPINAL CORD | Case report on the clinical results of a combined cellular therapy for chronic spinal cord injured patients | case report | 0 | 8 | 1 |
| VAQUERO J | 2018 | Spain | CYTOTHERAPY | Cell therapy with autologous mesenchymal stromal cells in post-traumatic syringomyelia | single arm | 2 | 6 | 6 |
| AL KANDARI S | 2018 | Kuwait | SPINAL CORD | Cell transplantation and clinical reality: Kuwait experience in persons with spinal cord injury | observational | 0 | 9 | / |
| CHERNYKH ER | 2006 | Russia | BULLETIN OF EXPERIMENTAL BIOLOGY AND MEDICINE | Characteristics of bone marrow cells under conditions of impaired innervation in patients with spinal trauma | single arm | 1 | 16 | / |
| LIU J | 2013 | China | CYTOTHERAPY | Clinical analysis of the treatment of spinal cord injury with umbilical cord mesenchymal stem cells | single arm | 1 | 22 | 1 |
| RAO YJ | 2013 | China | JOURNAL OF INTERNATIONAL MEDICAL RESEARCH | Clinical application of olfactory ensheathing cells in the treatment of spinal cord injury | single arm | 1 | 8 | 24 |
| ALBU S | 2021 | Spain | CYTOTHERAPY | Clinical effects of intrathecal administration of expanded Wharton jelly mesenchymal stromal cells in patients with chronic complete spinal cord injury: a randomized controlled study | RCT | 1/2a | 10 | 6 |
| KNOLLER N | 2005 | Isreal | JOURNAL OF NEUROSURGERY-SPINE | Clinical experience using incubated autologous macrophages as a treatment for complete spinal cord injury: Phase I study results | single arm | 1 | 8 | 6 |
| WU J | 2012 | China | CELL TRANSPLANTATION | Clinical Observation of Fetal Olfactory Ensheathing Glia Transplantation (OEGT) in Patients With Complete Chronic Spinal Cord Injury | single arm | 1/2a | 11 | 14 |
| CHENG HB | 2014 | China | JOURNAL OF TRANSLATIONAL MEDICINE | Clinical observation of umbilical cord mesenchymal stem cell transplantation in treatment for sequelae of thoracolumbar spinal cord injury | RCT | 1/2a | 10 | 6 |
| LEVI AD | 2018 | USA | JOURNAL OF NEUROTRAUMA | Clinical Outcomes from a Multi-Center Study of Human Neural Stem Cell Transplantation in Chronic Cervical Spinal Cord Injury | RCT | 2 | 12 | 12 |
| KARAMOUZIAN S | 2012 | Iran | CLINICAL NEUROLOGY AND NEUROSURGERY | Clinical safety and primary efficacy of bone marrow mesenchymal cell transplantation in subacute spinal cord injured patients | cohort | 1/2a | 11 | 12 |
| ZHAO YN | 2017 | China | CELL TRANSPLANTATION | Clinical Study of NeuroRegen Scaffold Combined With Human Mesenchymal Stem Cells for the Repair of Chronic Complete Spinal Cord Injury | single arm | 1 | 8 | 12 |
| SHIN JC | 2015 | South Korea | NEURAL PLASTICITY | Clinical Trial of Human Fetal Brain-Derived Neural Stem/Progenitor Cell Transplantation in Patients with Traumatic Cervical Spinal Cord Injury | cohort | 1/2a | 19 | 12 |
| DENG WS | 2020 | China | NEURAL REGENERATION RESEARCH | Collagen scaffold combined with human umbilical cord-mesenchymal stem cells transplantation for acute complete spinal cord injury | cohort | 1 | 20 | 12 |
| ORAEE-YAZDANI S | 2021 | Iran | STEM CELL RESEARCH & THERAPY | Combining cell therapy with human autologous Schwann cell and bone marrow-derived mesenchymal stem cell in patients with subacute complete spinal cord injury: safety considerations and possible outcomes | single arm | 1 | 11 | 12 |
| DAI G | 2014 | China | JOURNAL OF TRANSLATIONAL MEDICINE | Comparative analysis of curative effect of CT-guided stem cell transplantation and open surgical transplantation for sequelae of spinal cord injury | cohort | 1 | 18 | 6 |
| YOON SH | 2007 | South Korea | STEM CELLS | Complete spinal cord injury treatment using autologous bone marrow cell transplantation and bone marrow stimulation with granulocyte macrophage-colony stimulating factor: Phase I/II clinical trial | cohort | 1/2a | 35 | 10 |
| ORAEE-YAZDANI S | 2016 | Iran | SPINAL CORD | Co-transplantation of autologous bone marrow mesenchymal stem cells and Schwann cells through cerebral spinal fluid for the treatment of patients with chronic spinal cord injury: safety and possible outcome | single arm | 1 | 6 | 24 |
| CALLERA F | 2006 | Brazil | EXPERIMENTAL HEMATOLOGY | Delivery of autologous bone marrow precursor cells into the spinal cord via lumbar puncture technique in patients with spinal cord injury: A preliminary safety study | single arm | 1 | 10 | 3 |
| LARSON CA | 2013 | USA | JOURNAL OF SPINAL CORD MEDICINE | Effectiveness of intense, activity-based physical therapy for individuals with spinal cord injury in promoting motor and sensory recovery: Is olfactory mucosa autograft a factor? | cohort | 1 | 7 | 12 |
| RAO YJ | 2014 | China | GENETICS AND MOLECULAR RESEARCH | Effectiveness of olfactory ensheathing cell transplantation for treatment of spinal cord injury | single arm | 1 | 15 | / |
| AKHLAGHPASAND M | 2025 | Iran | CELL TRANSPLANTATION | Effects of Combined Intrathecal Mesenchymal Stem Cells and Schwann Cells Transplantation on Neuropathic Pain in Complete Spinal Cord Injury: A Phase II Randomized Active-Controlled Trial | RCT | 2 | 37 | 6 |
| FROLOV AA | 2012 | Russia | CELL TRANSPLANTATION | Effects of Hematopoietic Autologous Stem Cell Transplantation to the Chronically Injured Human Spinal Cord Evaluated by Motor and Somatosensory Evoked Potentials Methods | single arm | 1 | 20 | 12 |
| SAINI R | 2022 | India | JOURNAL OF CLINICAL NEUROSCIENCE | Efficacy and outcome of bone marrow derived stem cells transplanted via intramedullary route in acute complete spinal cord injury - A randomized placebo controlled trial | RCT | 2 | 7 | 6 |
| LEVI AD | 2018 | USA/Switzerland | NEUROSURGERY | Emerging Safety of Intramedullary Transplantation of Human Neural Stem Cells in Chronic Cervical and Thoracic Spinal Cord Injury | cohort | 2 | 29 | 12 |
| PAL R | 2009 | India | CYTOTHERAPY | Ex vivo-expanded autologous bone marrow-derived mesenchymal stromal cells in human spinal cord injury/paraplegia: a pilot clinical study | single arm | 1 | 30 | 12 |
| ABDELAZIZ OS | 2010 | Egypt | NEUROSURGERY QUARTERLY | Feasibility, Safety, and Efficacy of Directly Transplanting Autologous Adult Bone Marrow Stem Cells in Patients With Chronic Traumatic Dorsal Cord Injury A Pilot Clinical Study | single arm | 1 | 20 | 12 |
| SUGAI K | 2021 | Japan | REGENERATIVE THERAPY | First-in-human clinical trial of transplantation of iPSC-derived NS/PCs in subacute complete spinal cord injury: Study protocol | single arm | 1 | 4 | 12 |
| SHROFF G | 2016 | India | CTS-CLINICAL AND TRANSLATIONAL SCIENCE | Human Embryonic Stem Cell Therapy in Chronic Spinal Cord Injury: A Retrospective Study | single arm | 1 | 226 | 12 |
| DUMA C | 2019 | USA | MOLECULAR BIOLOGY REPORTS | Human intracerebroventricular (ICV) injection of autologous, non-engineered, adipose-derived stromal vascular fraction (ADSVF) for neurodegenerative disorders: results of a 3-year phase 1 study of 113 injections in 31 patients | single arm | 1 | 3 | 12 |
| GHOBRIAL GM | 2017 | USA | NEUROSURGERY | Human Neural Stem Cell Transplantation in Chronic Cervical Spinal Cord Injury: Functional Outcomes at 12 Months in a Phase II Clinical Trial | RCT | 2 | 4 | 12 |
| YAO LQ | 2013 | China | NEURAL REGENERATION RESEARCH | Human umbilical cord blood stem cell transplantation for the treatment of chronic spinal cord injury Electrophysiological changes and long-term efficacy | cohort | 1/2a | 25 | 12 |
| YANG Y | 2020 | China | NEURAL REGENERATION RESEARCH | Human umbilical cord mesenchymal stem cells to treat spinal cord injury in the early chronic phase: study protocol for a prospective, multicenter, randomized, placebo-controlled, single-blinded clinical trial | RCT | 2 | 44 | 12 |
| LAROCCA TF | 2017 | Brazil | CYTOTHERAPY | Image-guided percutaneous intralesional administration of mesenchymal stromal cells in subjects with chronic complete spinal cord injury: a pilot study | single arm | 1 | 5 | 6 |
| AKHLAGHPASAND M | 2025 | Iran | WORLD NEUROSURGERY | Improvement of Neurogenic Bladder Dysfunction Following Combined Cell Therapy with Mesenchymal Stem Cell and Schwann Cell in Spinal Cord Injury: A Randomized, Open-Label, Phase II Clinical Trial | RCT | 2 | 13 | 6 |
| VAQUERO J | 2018 | Spain | CYTOTHERAPY | Intrathecal administration of autologous mesenchymal stromal cells for spinal cord injury: Safety and efficacy of the 100/3 guideline | single arm | 2 | 11 | 10 |
| BYDON M | 2024 | USA | NATURE COMMUNICATIONS | Intrathecal delivery of adipose-derived mesenchymal stem cells in traumatic spinal cord injury: Phase I trial | single arm | 1 | 10 | 24 |
| HUR JW | 2016 | South Korea | JOURNAL OF SPINAL CORD MEDICINE | Intrathecal transplantation of autologous adipose-derived mesenchymal stem cells for treating spinal cord injury: A human trial | single arm | 1 | 14 | 10 |
| SHARMA A | 2020 | India | INTERNATIONAL JOURNAL OF HEALTH SCIENCES-IJHS | Intrathecal transplantation of autologous bone marrow mononuclear cells in patients with sub-acute and chronic spinal cord injury: An open-label study | single arm | 1 | 180 | 9 |
| HONMOU O | 2021 | Japan/USA | CLINICAL NEUROLOGY AND NEUROSURGERY | Intravenous infusion of auto serum-expanded autologous mesenchymal stem cells in spinal cord injury patients: 13 case series | case series | 2 | 13 | 6 |
| HIROTA R | 2024 | Japan/USA | JOURNAL OF CLINICAL MEDICINE | Intravenous Infusion of Autologous Mesenchymal Stem Cells Expanded in Auto Serum for Chronic Spinal Cord Injury Patients: A Case Series | case series | 1 | 7 | 6 |
| MARTIN JR | 2025 | USA | CELL REPORTS MEDICINE | Long-term clinical and safety outcomes from a single-site phase 1 study of neural stem cell transplantation for chronic thoracic spinal cord injury | single arm | 1 | 4 | 60 |
| TANG FW | 2021 | China | SCIENCE CHINA-LIFE SCIENCES | Long-term clinical observation of patients with acute and chronic complete spinal cord injury after transplantation of NeuroRegen scaffold | single arm | 1 | 66 | 24 |
| HUANG HY | 2012 | China | CELL TRANSPLANTATION | Long-Term Outcome of Olfactory Ensheathing Cell Therapy for Patients With Complete Chronic Spinal Cord Injury | single arm | 1 | 108 | 36 |
| RAO YJ | 2013 | China | CELL TRANSPLANTATION | Long-Term Outcome of Olfactory Ensheathing Cell Transplantation in Six Patients With Chronic Complete Spinal Cord Injury | single arm | 1 | 6 | 24 |
| PARK JH | 2012 | South Korea | NEUROSURGERY | Long-term Results of Spinal Cord Injury Therapy Using Mesenchymal Stem Cells Derived From Bone Marrow in Humans | single arm | 1 | 10 | 6 |
| KAPLAN N | 2025 | Turkey | WORLD JOURNAL OF STEM CELLS | Multiroute administration of Wharton's jelly mesenchymal stem cells in chronic complete spinal cord injury: A phase I safety and feasibility study | single arm | 1 | 6 | 12 |
| AKHLAGHPASAND M | 2025 | Iran | STEM CELL RESEARCH & THERAPY | Neurological, functional, and quality of life outcomes following combined mesenchymal stem cell and Schwann cell therapy in spinal cord injury: a 9-year experience | single arm | 1 | 106 | 12 |
| CHEN WG | 2020 | China | CELL TRANSPLANTATION | NeuroRegen Scaffolds Combined with Autologous Bone Marrow Mononuclear Cells for the Repair of Acute Complete Spinal Cord Injury: A 3-Year Clinical Study | single arm | 1 | 7 | 36 |
| LIMA C | 2006 | USA | JOURNAL OF SPINAL CORD MEDICINE | Olfactory mucosa autografts in human spinal cord injury: A pilot clinical study | single arm | 1 | 7 | 18 |
| LIMA C | 2010 | USA | NEUROREHABILITATION AND NEURAL REPAIR | Olfactory Mucosal Autografts and Rehabilitation for Chronic Traumatic Spinal Cord Injury | single arm | 1 | 20 | 12 |
| XIAO ZF | 2016 | China | SCIENCE CHINA-LIFE SCIENCES | One-year clinical study of NeuroRegen scaffold implantation following scar resection in complete chronic spinal cord injury patients | single arm | 1 | 5 | 12 |
| GANT KL | 2021 | USA | JOURNAL OF NEUROTRAUMA | Phase 1 Safety Trial of Autologous Human Schwann Cell Transplantation in Chronic Spinal Cord Injury | single arm | 1 | 8 | 6 |
| KAKABADZE Z | 2016 | USA | STEM CELLS INTERNATIONAL | Phase 1 Trial of Autologous Bone Marrow Stem Cell Transplantation in Patients with Spinal Cord Injury | single arm | 1 | 18 | 12 |
| ZHU H | 2016 | China/USA | CELL TRANSPLANTATION | Phase I-II Clinical Trial Assessing Safety and Efficacy of Umbilical Cord Blood Mononuclear Cell Transplant Therapy of Chronic Complete Spinal Cord Injury | single arm | 1/2a | 28 | 12 |
| YANG Y | 2021 | China | CYTOTHERAPY | Repeated subarachnoid administrations of allogeneic human umbilical cord mesenchymal stem cells for spinal cord injury: a phase 1/2 pilot study | single arm | 1/2a | 41 | 12 |
| VAQUERO J | 2017 | Spain | CYTOTHERAPY | Repeated subarachnoid administrations of autologous mesenchymal stromal cells supported in autologous plasma improve quality of life in patients suffering incomplete spinal cord injury | single arm | 2 | 10 | 12 |
| ZAMANI H | 2021 | Iran | SPINAL CORD | Safety and feasibility of autologous olfactory ensheathing cell and bone marrow mesenchymal stem cell co-transplantation in chronic human spinal cord injury: a clinical trial | single arm | 1 | 3 | 24 |
| SAINI R | 2022 | India | JOURNAL OF NEUROSURGERY-SPINE | Safety and feasibility of intramedullary injected bone marrow-derived mesenchymal stem cells in acute complete spinal cord injury: phase 1 trial | RCT | 1 | 7 | 6 |
| KODA M | 2024 | Japan | STEM CELL RESEARCH & THERAPY | Safety and feasibility of intravenous administration of a single dose of allogenic-Muse cells to treat human cervical traumatic spinal cord injury: a clinical trial | single arm | 1 | 10 | 12 |
| PAN K | 2019 | China | STEM CELLS INTERNATIONAL | Safety and Feasibility of Repeated Intrathecal Allogeneic Bone Marrow-Derived Mesenchymal Stromal Cells in Patients with Neurological Diseases | single arm | 1 | 4 | 6 |
| MENDONÇA MVP | 2014 | Brazil | STEM CELL RESEARCH & THERAPY | Safety and neurological assessments after autologous transplantation of bone marrow mesenchymal stem cells in subjects with chronic spinal cord injury | single arm | 1 | 14 | 6 |
| YAZDANI SO | 2013 | Iran | CYTOTHERAPY | Safety and possible outcome assessment of autologous Schwann cell and bone marrow mesenchymal stromal cell co-transplantation for treatment of patients with chronic spinal cord injury | single arm | 1 | 8 | 24 |
| AKHLAGHPASAND M | 2024 | Iran | STEM CELL RESEARCH & THERAPY | Safety and potential effects of intrathecal injection of allogeneic human umbilical cord mesenchymal stem cell-derived exosomes in complete subacute spinal cord injury: a first-in-human, single-arm, open-label, phase I clinical trial | single arm | 1 | 9 | 12 |
| AWIDI A | 2024 | Jordan | CYTOTHERAPY | Safety and potential efficacy of expanded mesenchymal stromal cells of bone marrow and umbilical cord origins in patients with chronic spinal cord injuries: a phase I/II study | RCT | 1/2a | 20 | 24-36 |
| YANG WZ | 2010 | China/USA | JOURNAL OF TRANSLATIONAL MEDICINE | Safety evaluation of allogeneic umbilical cord blood mononuclear cell therapy for degenerative conditions | single arm | 1 | 42 | 1 |
| ANDERSON KD | 2017 | USA | JOURNAL OF NEUROTRAUMA | Safety of Autologous Human Schwann Cell Transplantation in Subacute Thoracic Spinal Cord Injury | single arm | 1 | 6 | 12 |
| SABERI H | 2011 | UK | JOURNAL OF NEUROSURGERY-SPINE | Safety of intramedullary Schwann cell transplantation for postrehabilitation spinal cord injuries: 2-year follow-up of 33 cases | single arm | 1 | 33 | 24 |
| RA JC | 2011 | South Korea | STEM CELLS AND DEVELOPMENT | Safety of Intravenous Infusion of Human Adipose Tissue-Derived Mesenchymal Stem Cells in Animals and Humans | single arm | 1 | 8 | 3 |
| XIAO ZF | 2018 | China | CELL TRANSPLANTATION | Significant Improvement of Acute Complete Spinal Cord Injury Patients Diagnosed by a Combined Criteria Implanted with NeuroRegen Scaffolds and Mesenchymal Stem Cells | single arm | 1 | 2 | 12 |
| CRISTANTE AF | 2009 | Brazil | SPINAL CORD | Stem cells in the treatment of chronic spinal cord injury: evaluation of somatosensitive evoked potentials in 39 patients | case series | 0 | 39 | 30 |
| ZHAO Y | 2021 | China | INTERNATIONAL JOURNAL OF GENERAL MEDICINE | Study of the Diffusion Tensor Imaging for Preclinical Therapeutic Efficacy of Umbilical Cord Mesenchymal Stem Cell Transplantation in the Treatment of Spinal Cord Injury | cohort | 1 | 7 | 6 |
| SMIRNOV VA | 2022 | Russia | WORLD NEUROSURGERY | Systemic Administration of Allogeneic Cord Blood Mononuclear Cells in Adults with Severe Acute Contusion Spinal Cord Injury: Phase 1/2a Pilot Clinical Study-Safety and Primary Efficacy Evaluation | single arm | 1/2a | 10 | 12 |
| MCKENNA SL | 2022 | USA | JOURNAL OF NEUROSURGERY-SPINE | Ten-year safety of pluripotent stem cell transplantation in acute thoracic spinal cord injury | single arm | 1 | 5 | 60 |
| CURT A | 2020 | Switzerland/Canada | NEUROREHABILITATION AND NEURAL REPAIR | The Damaged Spinal Cord Is a Suitable Target for Stem Cell Transplantation | single arm | 1/2a | 12 | 72 |
| YANG YL | 2020 | China | INTERNATIONAL JOURNAL OF CLINICAL AND EXPERIMENTAL MEDICINE | Therapeutic effect of mesenchymal stem cell in spinal cord injury | RCT | 1/2a | 34 | 6 |
| ZHOU XH | 2012 | China | CELL TRANSPLANTATION | Transplantation of Autologous Activated Schwann Cells in the Treatment of Spinal Cord Injury: Six Cases, More Than Five Years of Follow-up | case series | 0 | 6 | 60 |
| DAI GH | 2013 | China | BRAIN RESEARCH | Transplantation of autologous bone marrow mesenchymal stem cells in the treatment of complete and chronic cervical spinal cord injury | RCT | 1/2a | 20 | 6 |
| MACÊDO CT | 2024 | Brazil | FRONTIERS IN MEDICINE | Transplantation of autologous mesenchymal stromal cells in complete cervical spinal cord injury: a pilot study | single arm | 1 | 6 | 12 |
| TABAKOW P | 2013 | Poland | CELL TRANSPLANTATION | Transplantation of Autologous Olfactory Ensheathing Cells in Complete Human Spinal Cord Injury | cohort | 1 | 3 | 12 |
| AL-ZOUBI A | 2015 | USA | CELL TRANSPLANTATION | Transplantation of Purified Autologous Leukapheresis-Derived CD34+ and CD133+ Stem Cells for Patients With Chronic Spinal Cord Injuries: Long-Term Evaluation of Safety and Efficacy | single arm | 1 | 19 | 51 |
| DEDA H | 2008 | Turkey | CYTOTHERAPY | Treatment of chronic spinal cord injured patients with autologous bone marrow-derived hematopoietic stem cell transplantation:: 1-year follow-up | single arm | 1 | 9 | 12 |
| SABERI H | 2008 | Iran | NEUROSCIENCE LETTERS | Treatment of chronic thoracic spinal cord injury patients with autologous Schwann cell transplantation: An interim report on safety considerations and possible outcomes | single arm | 1 | 4 | 12 |
| PARK HC | 2005 | South Korea | TISSUE ENGINEERING | Treatment of complete spinal cord injury patients by autologous bone marrow cell transplantation and administration of granulocyte-macrophage colony stimulating factor | single arm | 1 | 6 | 6-18 |
| BURGOS-GUTIÉRREZ C | 2022 | Spain | JOURNAL OF SPINAL CORD MEDICINE | Treatment of pressure ulcers in patients with spinal cord injury: Conventional surgery vs. cellular therapy | cohort | 1/2a | 149 | 12 |
| SARASÚA JG | 2011 | Spain | JOURNAL OF SPINAL CORD MEDICINE | Treatment of pressure ulcers with autologous bone marrow nuclear cells in patients with spinal cord injury | single arm | 1 | 22 | 12 |
| JEON SR | 2010 | South Korea | TISSUE ENGINEERING AND REGENERATIVE MEDICINE | Treatment of Spinal Cord Injury with Bone Marrow-Derived, Cultured Autologous Mesenchymal Stem Cells | single arm | 1 | 10 | 6 |
| MIAO XY | 2015 | China | INDIAN JOURNAL OF BIOCHEMISTRY & BIOPHYSICS | Umbilical cord mesenchymal stem cells in neurological disorders: A clinical study | single arm | 1 | 30 | 12 |

This table shows basic information of involved articles and the design of the trials.

**Design:** The type of trial design. **Phase:** Clinical trial phase as defined by the original author(s). **Subjects:** The number of patients enrolled in the trial. **Follow-Up:** Duration of follow-up investigation. Presented as the total number of months, or the overall interval of follow-up time.

**Supplementary Table 3**: Characteristics of Cell Therapy Treatment

| **First Author** | **Year** | **Cell Type** | **Cell Source** | **Delivery** | **Cell Dose**  **(10^7^)** | **Delivery Times** |
| --- | --- | --- | --- | --- | --- | --- |
| JIANG PC | 2013 | mesenchymal stromal cell | bone marrow (autologous) | IT+IL | 10 | 1 |
| CURTIS E | 2018 | neural stem cell | spinal cord (allogeneic) | IL | 0.12 | 1 |
| FESSLER RG | 2022 | oligodendrocyte progenitor cell | human embryonic stem cell (allogeneic) | IL | 0.2/1/2 | 1 |
| OH SK | 2016 | mesenchymal stromal cell | bone marrow (autologous) | IT+IL | 4.8 | 1 |
| IWATSUKI K | 2016 | olfactory ensheathing cell, neural stem cell | olfactory mucosa (autologous) | IL | / | 1 |
| CHEN L | 2015 | olfactory ensheathing cell,Schwann cell | fetal olfactory bulb, human fetal sciatic nerve (allogeneic) | IL | 0.1 | 1 |
| GEFFNER LF | 2008 | mononuclear cell | bone marrow (autologous) | IT+IL+IV | 40 | 1 |
| SHARMA A | 2012 | mononuclear cell | bone marrow (autologous) | IT | 0.1/kg | 1 |
| SAITO F | 2012 | mesenchymal stromal cell | bone marrow (autologous) | IT | 3-5 | 1 |
| VAQUERO J | 2016 | mesenchymal stromal cell | bone marrow (autologous) | IT+IL | 0.3 | 3 |
| ATTAR A | 2011 | mononuclear cell | bone marrow (autologous) | IL | 2-62 | 1 |
| CHERNYKH ER | 2007 | mononuclear cell | bone marrow (autologous) | IL+IV | 3.6 | 1 |
| CHHABRA HS | 2016 | mononuclear cell | bone marrow (autologous) | IT+IL | 20 | 2 |
| KUMAR AA | 2009 | mononuclear cell | bone marrow (autologous) | IT | 37.2 | 1 |
| SYKOVÁ E | 2006 | mononuclear cell | bone marrow (autologous) | IA+IV | 1040 | 1 |
| EL-KHEIR WA | 2014 | mononuclear cell | bone marrow (autologous) | IT | 0.2/kg | 4 |
| LAMMERTSE DP | 2012 | macrophage | skin, blood (autologous) | IL | 0.15 | 1 |
| BHANOT Y | 2011 | mesenchymal stromal cell | bone marrow (autologous) | IT+IL | 0.1-0.4/kg | 3 |
| SATTI HS | 2016 | mesenchymal stromal cell | bone marrow (autologous) | IT | 0.12/kg | 2-3 |
| CHHABRA HS | 2009 | olfactory ensheathing cell, neural stem cell | olfactory mucosa (autologous) | IL | 1.1 | 1 |
| MACKAY-SIM A | 2008 | olfactory ensheathing cell | olfactory mucosa (autologous) | IL | 1.2-2.8 | 1 |
| FÉRON F | 2005 | olfactory ensheathing cell | olfactory mucosa (autologous) | IL | 1.2-2.8 | 1 |
| WANG S | 2016 | olfactory ensheathing cell | olfactory mucosa (autologous) | IL | / | 1 |
| SONG H | 2020 | mesenchymal stromal cell | bone marrow (autologous) | IT | 1 | 1 |
| SUZUKI Y | 2014 | mononuclear cell | bone marrow (autologous) | IT | 41 | 1 |
| AMR SM | 2014 | mesenchymal stromal cell, scaffold | bone marrow (autologous) | IL | / | 1 |
| KISHK NA | 2010 | mononuclear cell | bone marrow (autologous) | IT | 0.5-1/kg | 6 |
| MOVIGLIA GA | 2009 | mononuclear cell, neural stem cell, effector T cell | bone marrow (autologous) | IA+IV | / | 3 |
| VAQUERO J | 2018 | mesenchymal stromal cell | bone marrow (autologous) | IL | 30 | 1 |
| AL KANDARI S | 2018 | / | / (autologous and allogeneic) | / | / | 1-4 |
| CHERNYKH ER | 2006 | mononuclear cell, T cell | bone marrow (autologous) | / | 1.1 | / |
| LIU J | 2013 | mesenchymal stromal cell | umbilical cord (allogeneic) | IT | 0.1/kg | 4 |
| RAO YJ | 2013 | olfactory ensheathing cell | olfactory mucosa (autologous) | IL | 0.1 | 8 |
| ALBU S | 2021 | mesenchymal stromal cell | umbilical cord (allogeneic) | IT | 1 | 1 |
| KNOLLER N | 2005 | macrophage | skin, blood (autologous) | IL | 4 | 1 |
| WU J | 2012 | olfactory ensheathing cell | olfactory bulb (allogeneic) | IL | 0.05 | 1 |
| CHENG HB | 2014 | mesenchymal stromal cell | umbilical cord (allogeneic) | IL | 2 | 2 |
| LEVI AD | 2018 | neural stem cell | brain, spinal cord (allogeneic) | IL | 1.5-4 | 1 |
| KARAMOUZIAN S | 2012 | mononuclear cell | bone marrow (autologous) | IT | 0.1 | 1 |
| ZHAO YN | 2017 | mesenchymal stromal cell, scaffold | umbilical cord (allogeneic) | IL | 4 | 1 |
| SHIN JC | 2015 | neural stem cell | brain (allogeneic) | IL | 2.5 | 1 |
| DENG WS | 2020 | mesenchymal stromal cell, scaffold | umbilical cord (allogeneic) | IL | 4 | 1 |
| ORAEE-YAZDANI S | 2021 | mononuclear cell,schwann cell | bone marrow, sural nerve (autologous) | IT | 10 | 1 |
| DAI G | 2014 | mesenchymal stromal cell | umbilical cord (allogeneic) | IL | 2 | 2 |
| YOON SH | 2007 | mononuclear cell | bone marrow (autologous) | IL | 20 | 1 |
| ORAEE-YAZDANI S | 2016 | mesenchymal stromal cell, schwann cell | bone marrow, nerve (autologous) | IT | 0.4 | 2 |
| CALLERA F | 2006 | mononuclear cell | bone marrow (autologous) | IT | 10 | 1 |
| LARSON CA | 2013 | olfactory ensheathing cell | olfactory mucosa (autologous) | IL | / | 1 |
| RAO YJ | 2014 | olfactory ensheathing cell | olfactory bulb (allogeneic) | IL | 0.2-0.5 | 1 |
| AKHLAGHPASAND M | 2025 | mesenchymal stromal cel, Schwann cells | bone marrow, sural nerve (autologous) | IT | 0.6 | 1 |
| FROLOV AA | 2012 | hematopoietic stem cell | blood (autologous) | IT | 1500-2000 | 4 |
| SAINI R | 2022 | mononuclear cell | bone marrow (autologous) | IL | 20 | 1 |
| LEVI AD | 2018 | neural stem cell | brain, spinal cord (allogeneic) | IL | 1.5-4 | 1 |
| PAL R | 2009 | mesenchymal stromal cell | bone marrow (autologous) | IT | 0.1/kg | 1 |
| ABDELAZIZ OS | 2010 | mononuclear cell | bone marrow (autologous) | IL | 0.5/kg | 1 |
| SUGAI K | 2021 | iPSC-derived neural stem cell | blood (allogeneic) | IL | 0.2 | 1 |
| SHROFF G | 2016 | embryonic stem cell (multipotent stem cell) | IVF (allogeneic) | IT+IV | / | 10 |
| DUMA C | 2019 | mononuclear cell | adipose tissue (autologous) | intraventricular | / | 1 |
| GHOBRIAL GM | 2017 | neural stem cell | brain, spinal cord (allogeneic) | IL | 1.5-4 | 1 |
| YAO LQ | 2013 | mononuclear cell | umbilical cord (allogeneic) | IT+IV | 1-3 | 1 |
| YANG Y | 2020 | mesenchymal stromal cell | umbilical cord (allogeneic) | IT | 0.1/kg | 4 |
| LAROCCA TF | 2017 | mesenchymal stromal cell | bone marrow (autologous) | IL | 2 | 1 |
| AKHLAGHPASAND M | 2025 | mesenchymal stromal cell, schwann cell | bone marrow, sural nerve (autologous) | IT | 6 | 1 |
| VAQUERO J | 2018 | mesenchymal stromal cell | bone marrow (autologous) | IT | 10 | 3 |
| BYDON M | 2024 | mesenchymal stromal cell | adipose tissue (autologous) | IT | 10 | 1 |
| HUR JW | 2016 | mesenchymal stromal cell | adipose tissue (autologous) | IT | 3 | 3 |
| SHARMA A | 2020 | mononuclear cell | bone marrow (autologous) | IT | 10 | 1 or more than 1 |
| HONMOU O | 2021 | mesenchymal stromal cell | bone marrow (autologous) | IV | 10 | 1 |
| HIROTA R | 2024 | mesenchymal stromal cell | bone marrow (autologous) | IV | 13 | 1 |
| MARTIN JR | 2025 | neural stem cell | spinal cord (allogeneic) | IL | 0.12 | 1 |
| TANG FW | 2021 | mesenchymal stromal cell, scaffold | umbilical cord, bone marrow (allogeneic) | IL | 4, 100 | 1 |
| HUANG HY | 2012 | olfactory ensheathing cell | olfactory bulb (allogeneic) | IL | / | 1 |
| RAO YJ | 2013 | olfactory ensheathing cell | olfactory bulb (allogeneic) | IL | 0.2 | 1 |
| PARK JH | 2012 | mesenchymal stromal cell | bone marrow (autologous) | IT+IL | 5 | 2 |
| KAPLAN N | 2025 | mesenchymal stromal cell | umbilical cord (allogeneic) | IT+IV+IM | 0.3/kg | 4 |
| AKHLAGHPASAND M | 2025 | mesenchymal stromal cell, schwann cell | bone marrow, sural nerve (autologous) | IT | 0.6 | 1 |
| CHEN WG | 2020 | mononuclear cell, scaffold | bone marrow (autologous) | IL | 100 | 1 |
| LIMA C | 2006 | olfactory ensheathing cell | olfactory mucosa (autologous) | IL | / | 1 |
| LIMA C | 2010 | olfactory ensheathing cell | olfactory mucosa (autologous) | IL | / | 1 |
| XIAO ZF | 2016 | mononuclear cell, scaffold | bone marrow (autologous) | IL | 100 | 1 |
| GANT KL | 2021 | schwann cell | sural nerve (autologous) | IL | 2.3-7 | 1 |
| KAKABADZE Z | 2016 | mononuclear cell | bone marrow (autologous) | IT | 75 | 1 |
| ZHU H | 2016 | mononuclear cell | umbilical cord (allogeneic) | IL | 0.16-0.64 | 1 |
| YANG Y | 2021 | mesenchymal stromal cell | umbilical cord (allogeneic) | IT | 0.1/kg | 4 |
| VAQUERO J | 2017 | mesenchymal stromal cell | bone marrow (autologous) | IT | 3 | 4 |
| ZAMANI H | 2021 | mesenchymal stromal cell, olfactory ensheathing cell | bone marrow, olfactory mucosa (autologous) | IT | 3 | 1 |
| SAINI R | 2022 | mesenchymal stromal cell | bone marrow (autologous) | IL | 20 | 1 |
| KODA M | 2024 | Muse cell (multipotent stem cell) | bone marrow (allogeneic) | IV | 1.5 | 1 |
| PAN K | 2019 | mesenchymal stromal cell | bone marrow (allogeneic) | IT | 0.1/kg | 4 |
| MENDONÇA MVP | 2014 | mesenchymal stromal cell | bone marrow (autologous) | IL | 1 | 1 |
| YAZDANI SO | 2013 | mesenchymal stromal cell, schwann cell | bone marrow, sural nerve (autologous) | IL | 0.1 | 1 |
| AKHLAGHPASAND M | 2024 | exosomes | umbilical cord (allogeneic) | IT | / | 1 |
| AWIDI A | 2024 | mesenchymal stromal cell | bone marrow, umbilical cord (autologous, allogeneic) | IT | 10 | 3 |
| YANG WZ | 2010 | mesenchymal stromal cell | umbilical cord (allogeneic) | IT+IV | 1-3 | 4-5 |
| ANDERSON KD | 2017 | schwann cell | sural nerve (autologous) | IL | 20 | 1 |
| SABERI H | 2011 | schwann cell | sural nerve (autologous) | IL | 0.3 | 1 |
| RA JC | 2011 | mesenchymal stromal cell | adipose tissue (autologous) | IV | 0.5-25/kg | 1 |
| XIAO ZF | 2018 | mesenchymal stromal cell, scaffold | umbilical cord (allogeneic) | IL | 4 | 1 |
| CRISTANTE AF | 2009 | mononuclear cell | blood (autologous) | IA | 0.26 | 1 |
| ZHAO Y | 2021 | mesenchymal stromal cell | umbilical cord (allogeneic) | IT | 5 | 4 |
| SMIRNOV VA | 2022 | mononuclear cell | umbilical cord (allogeneic) | IV | 1.48/kg | 4 |
| MCKENNA SL | 2022 | oligodendrocyte progenitor cell | human embryonic stem cell (allogeneic) | IL | 0.2 | 1 |
| CURT A | 2020 | neural stem cell | brain, spinal cord (allogeneic) | IL | 2 | 1 |
| YANG YL | 2020 | mesenchymal stromal cell | bone marrow (autologous) | IL | 2 | 1 |
| ZHOU XH | 2012 | schwann cell | sural nerve (autologous) | IL | 0.4-0.6 | 1 |
| DAI GH | 2013 | mesenchymal stromal cell | bone marrow (autologous) | IL | 2 | 1 |
| MACÊDO CT | 2024 | mesenchymal stromal cell | bone marrow (autologous) | IT+IL | 5 | 2 |
| TABAKOW P | 2013 | olfactory ensheathing cell, fibroblast | olfactory mucosa (autologous) | IL | 0.6 | 1 |
| AL-ZOUBI A | 2015 | mononuclear cell | blood (autologous) | IT+IL | 7.6 | 1 |
| DEDA H | 2008 | hematopoietic stem cell | bone marrow (autologous) | IT+IL+IV | 20 | 1 |
| SABERI H | 2008 | schwann cell | sural nerve (autologous) | IL | 0.3 | 1 |
| PARK HC | 2005 | mononuclear cell | bone marrow (autologous) | IL | 200 | 1 |
| BURGOS-GUTIÉRREZ C | 2022 | mononuclear cell | bone marrow (autologous) | Intra-ulcer | / | 1 |
| SARASÚA JG | 2011 | mononuclear cell | bone marrow (autologous) | Intra-ulcer | 26.5 | 1 |
| JEON SR | 2010 | mesenchymal stromal cell | bone marrow (autologous) | IT+IL | 4.8 | 3 |
| MIAO XY | 2015 | mesenchymal stromal cell | umbilical cord (allogeneic) | IT | / | 4-6 |

This table shows characteristics of cell therapy treatment of the involved articles. Missing values are due to insufficient data.

**Cell type:** The type(s) of cells that are administered by researchers. **Cell source:** The origin of cells. Shown as origin of tissue and individual (allogeneic or autologous). **Delivery:** Route of administration. Including intrathecal(IT), intralesion(IL), intravenous(IV), intramuscular(IM) and other methods. **Cell dose:** Amount of cell used (×10^7^). Shown as total number of cells, range of dosage, or dosage per weight unit. **Delivery times:** The frequency of the cell treatment. Presented as the total number of times or range of times.

**Supplementary Table 4:** General Characteristics of Patients

| **First Author** | **Year** | **Gender** | **Age** | **Injury Site** | **Severity** | **Duration** |
| --- | --- | --- | --- | --- | --- | --- |
| JIANG PC | 2013 | 13M/7F | 41.10±15.64 | 4C,11T,4L,1S | 8A,4B,8C | >6m(except 1) |
| CURTIS E | 2018 | 3M/1F | 29.50±5.32 | 4T | 4A | 372d(range 175d-533d) |
| FESSLER RG | 2022 | 21M/4F | 31.80±14.80 | 25C | 15A,10B | 14d-30d |
| OH SK | 2016 | 15M/1F | 40.87±13.56 | 16C | 1A, 15B | >1y |
| IWATSUKI K | 2016 | 6M/2F | 31.50±7.80 | 8T | 7A, 1B | 108.5m±105m |
| CHEN L | 2015 | 4M/1F | 28.60±8.23 | 5C | 5A | 99.20m±42.24m |
| GEFFNER LF | 2008 | 7F/1M | 33.75±6.58 | 8T | 5A, 1B, 2C | 61.14m±88.54m |
| SHARMA A | 2012 |  | 8.8 |  |  |  |
| SAITO F | 2012 | 5M | 42.60±12.03 | 5C | 3A, 1B, 1C | 13.00d±3.24d |
| VAQUERO J | 2016 | 9M/3F | 40.50±8.75 | 12T | 12A | 13.86y±9.36y |
| ATTAR A | 2011 | 2M/2F | 22.00±2.94 | 1C,3T | 4A | <14d |
| CHERNYKH ER | 2007 | 14M/4F | 31.6 | 12C,2T,4L |  | >6m |
| CHHABRA HS | 2016 | 11M/3F | 24.86±5.84 | 14T | 6A, 8B | 10d-14d |
| KUMAR AA | 2009 |  |  |  |  |  |
| SYKOVÁ E | 2006 | 16M/4F | 30.05±7.46 | 12C,8T | 15A, 4B, 1C | 10d-18m |
| EL-KHEIR WA | 2014 |  |  | 10C, 40T | 15A, 35B | 18.76m±5.71m |
| LAMMERTSE DP | 2012 | 27M/6F | 27.4±11.0 | 15C,18T | 33A | <14d |
| BHANOT Y | 2011 | 10M/3F | 32.23±9.08 | 5C,8T | 13A | 34.08m±45.34m |
| SATTI HS | 2016 | 9M | 31.56±4.77 | 9T | 9A | 23.11m±23.35m |
| CHHABRA HS | 2009 | 5M | 27.20±6.38 | 1C,4T | 5A | 48.20m±28.44m |
| MACKAY-SIM A | 2008 | 3M | 33.00±11.79 | 3T | 3A | 18m-32m |
| FÉRON F | 2005 | 3M | 33.00±11.79 | 3T | 3A | 18m-32m |
| WANG S | 2016 | 7M/1F | 29.25±7.46 | 8T | 8A | 27.25m±19.14m |
| SONG H | 2020 | 12M/6F | 41.2±2.3 | 10C,5T,3L |  | <7d |
| SUZUKI Y | 2014 | 10M | 30.70±10.98 | 6T, 4C | 5A,5B | 129.5d±98.01d |
| AMR SM | 2014 | 12M/2F | 28.64±9.76 | 14T | 14A | 20.64m±20.55m//23m |
| KISHK NA | 2010 | 36M/7F | 31.7±10.4 | 6C,37T | 40A, 3C | 3.6y±2.5y |
| MOVIGLIA GA | 2009 |  |  |  | 8A |  |
| VAQUERO J | 2018 | 6M | 39.0±7.6 | 5T,1L | 3A,2B,1D | 13.73y±8.65y |
| AL KANDARI S | 2018 | 8M/1F | 23.33±3.39 | 3C,6A | 6A,1B,2C | 42m±38.2m |
| CHERNYKH ER | 2006 |  |  |  |  |  |
| LIU J | 2013 | 17M/5F | 32.64±9.85 | 6C,9T,7L | 6A,16BCD | 55.90m±61.37m |
| RAO YJ | 2013 | 5M/3F | 33.0±10.4 | 8C | 3A,3B,2C | 10.62m±2.50m |
| ALBU S | 2021 | 7M/3F | 32.70±8.19 | 10T | 10A | 31.90m±12.74m |
| KNOLLER N | 2005 | 7M/1F | 26.25±7.59 | 1C,9T | 8A | 12.25d±2.05d |
| WU J | 2012 | 9M/2F | 35.18±10.67 | 5C,6T | 11A | 3.54y±1.23y |
| CHENG HB | 2014 |  | 35.30±8.23 | 10T | 10A | 21.46m±12.96m |
| LEVI AD | 2018 | 11M/1F | 28.33±7.06 | 12C | 3A,9B | 1.04y±0.48y |
| KARAMOUZIAN S | 2012 | 6M/5F | 33.18±8.9 | 11T | 11A | 27.3d±8.4d |
| ZHAO YN | 2017 | 7M/1F | 31.5±5.32 | 4C,4T | 8A | 13.38m±10.15m |
| SHIN JC | 2015 | 16M/3F | 37.21±12.70 | 19C | 17A,2B | 63.42d±54.11d |
| DENG WS | 2020 | 5M/15F | 33.70±9.03 | 20C | 20A | 12.45d±5.74d |
| ORAEE-YAZDANI S | 2021 | 9M/2F | 29.09±9.41 | 4C, 7T | 11A | 5.27m±2.18m |
| DAI G | 2014 | 16M/2F | 36.00±9.68 | 10C, 4T | 12A,4B,2C | 18.67m±7.68m |
| YOON SH | 2007 | 29M/6F | 38.92 | 23C, 12T | 35A | <2w(17), 2-8w(6), >8w(12) |
| ORAEE-YAZDANI S | 2016 | 4M/2F | 33.33±8.66 | 1C,5T | 6A | 38.10m±15.28m |
| CALLERA F | 2006 |  | 24 |  |  | 3y |
| LARSON CA | 2013 | 7M | 30.3±9.1 | 7CT | 5A,1B,1C | 2.9y±1.9y |
| RAO YJ | 2014 | 12M/3F | 18-56 | 6C,2T,7L |  | 6m-48m |
| AKHLAGHPASAND M | 2025 | 31M/6F | 30.3±8.5 | 12C,25T | 37A | 15.9m±14.9m |
| FROLOV AA | 2012 | 15M/5F | 32.41±11.67 | 20C |  | 3.39y±3.11y |
| SAINI R | 2022 | 6M/1F | 28.1±18.0 |  | 7A | <21d |
| LEVI AD | 2018 | 27M/2F | 29.41±9.07 | 17C,12T | 11A,18B | 20.59m±17.33m |
| PAL R | 2009 | 27M/3F | 33.2±10.92 | 7C,22T | 24A,6C | <6m(20), >6m(10) |
| ABDELAZIZ OS | 2010 | 18M/2F | 26.55±11.18 | 20T | 10A,5B,5C | 27.15m±28.61m |
| SUGAI K | 2021 |  |  |  | 4A |  |
| SHROFF G | 2016 | 167M/59F | 28(20-34) |  | 153A,32B,36C,5D | 73d |
| DUMA C | 2019 |  |  |  |  |  |
| GHOBRIAL GM | 2017 | 5M | 24.3±3.78 | 4C | 1A,3B |  |
| YAO LQ | 2013 | 16M/9F | 36.7±3.8 | 5C,11T,9L |  | 11.4m±3.6m |
| YANG Y | 2020 |  |  |  |  |  |
| LAROCCA TF | 2017 | 5M | 44.80±6.69 | 5T | 5A | 70.2m±30.61m |
| AKHLAGHPASAND M | 2025 | 10M/3F | 35.0±8.8 | 4C,9T | 13A | 15.69m±15.53m |
| VAQUERO J | 2018 | 7M/4F | 44.91±10.17 | 4C,4T,3L | 3A,4B,3C,1D | 13.65y±14.79y |
| BYDON M | 2024 | 8M/2F | 34.6 | 4C,6T | 5A,2B,3C | 12.00m±4.22m |
| HUR JW | 2016 | 12M/2F | 41.9±15.5 | 5C,6T,1L | 10A,1B,1D | 13.7m±7.6m |
| SHARMA A | 2020 | 160M/20F | 32.01 | 63C,117T | 138A,28B,10C,3D | <12m(60), >12m(120) |
| HONMOU O | 2021 | 12M/1F | 46.92±14.65 | 13C | 6A,2B,5C | 49.15d±3.78d |
| HIROTA R | 2024 | 5M/2F | 39.57±13.11 | 7C | 2C,5D | 8.57y±9.93y |
| MARTIN JR | 2025 | 3M/1F | 32.00±4.76 | 4T | 4A | 1y-2y |
| TANG FW | 2021 |  | 38.52 | 22C,44T | 66A | <14d(15), >2m(51) |
| HUANG HY | 2012 | 84M/24F | 34.29±10.39 | 51C,42T,15L | 108A | 3.48y±4.73y |
| RAO YJ | 2013 | 4M/2F | 39.67±9.81 | 2C,4T | 6A | 18.83m±5.78m |
| PARK JH | 2012 | 8M/2F | 46.00±8.51 | 10C | 4A,6B | 40.2m±40.3m |
| KAPLAN N | 2025 | 5M/1F | 27.50±7.31 | 6T | 6A | 38.00m±54.25m |
| AKHLAGHPASAND M | 2025 | 87M/17F | 31.5±10.0 | 32C,74T | 106A | 23.9m±32.1m |
| CHEN WG | 2020 | 7M | 46.57±10.47 | 7T | 7A | 8.71d±8.30d |
| LIMA C | 2006 | 4M/3F | 23.4±5.4 | 3C,4T | 7A | 33.43m±24.95m |
| LIMA C | 2010 | 17M/3F | 28.10±5.73 | 12C,8T | 15A,5B | 49.35m±42.18m |
| XIAO ZF | 2016 | 4M/1F | 43.20±14.00 | 1C,4T | 5A | 13.00m±11.49m |
| GANT KL | 2021 | 6M/2F | 33.25±13.38 | 4C,4T | 4A,2B,2C | 6.25y±4.77y |
| KAKABADZE Z | 2016 | 13M/5F | 37.11±16.63 | 12T,5L,1S | 10A,5B,3C | 12.11m±4.71m |
| ZHU H | 2016 | 23M/5F | 38.14±10.41 | 9C,19T | 28A | 8.81y±6.28y |
| YANG Y | 2021 | 33M/8F | 38.1±11.0 | 24C,7T,10TL | 13A,28BCD | 45.15m±59.78m |
| VAQUERO J | 2017 | 8M/2F | 42.20±9.30 | 5C,2T,3L | 4B,5C,1D | 14.21y±9.88y |
| ZAMANI H | 2021 | 2M/1F | 39.67±22.81 | 3T | 3A | 50.0m±26.1m |
| SAINI R | 2022 | 12M/2F | 28.1±18.0 | 6C/7T/1L | 7A | <1w |
| KODA M | 2024 | 8M/2F | 49.3(27-67) | 10C |  | 12d-16d |
| PAN K | 2019 | 4M | 42.25±17.46 |  |  | 5.25m±4.99m |
| MENDONÇA MVP | 2014 | 10M/4F | 35.7±9.9 | 13T,1L | 14A | 61.79m±51.53m |
| YAZDANI SO | 2013 | 4M/4F | 27.9±9.9 | 1C,7T | 8A | 13-63m |
| AKHLAGHPASAND M | 2024 | 5M/4F | 33.56±7.62 | 3C,6T | 9A | 2.78m±1.33m |
| AWIDI A | 2024 | 17M/3F | 37.12 | 7C,13T | 16A,4B | 76.52d |
| YANG WZ | 2010 |  |  |  |  |  |
| ANDERSON KD | 2017 | 6M | 31.83±7.25 | 6T | 6A | 1m |
| SABERI H | 2011 | 22M/11F | 32.9 | 9C,24T | 16A,17B | 4.1y |
| RA JC | 2011 | 8M | 23-54 |  |  | 1.07y-7.88y |
| XIAO ZF | 2018 | 2M | 29.00±1.41 | 1C,1T | 2A | 4.50d±4.95d |
| CRISTANTE AF | 2009 | 28M/11F |  | 14C,25T | 39A | >2y |
| ZHAO Y | 2021 | 6M/1F | 32.43±9.59 | 3C,4T | 6A,1C | 7.43m±6.08m |
| SMIRNOV VA | 2022 | 9M/1F | 41.90±15.51 | 4C,5T,1L | 6A,4B | 2.6d±0.52d |
| MCKENNA SL | 2022 | 4M/1F | 21-32 | 5T | 5A | 7-14d |
| CURT A | 2020 | 12M | 30.9±10.0 | 12T | 7A,5B | 10.0m±5.0m |
| YANG YL | 2020 | 27M/7F | 35.29±8.04 | 23C,11T | 22A,7B,5C | 20.67m±5.31m |
| ZHOU XH | 2012 | 5M/1F | 27.17±18.58 | 1C,5T | 3A,2B,1C | 5.67m±7.33m |
| DAI GH | 2013 | 14M/6F | 34.7±8.9 | 20C | 20A | 51.9m±18.3 |
| MACÊDO CT | 2024 | 5M/1F | 39.83±9.58 | 6C | 6A | 128.5m±64.1m |
| TABAKOW P | 2013 | 3M | 24.67±1.54 | 3T | 3A | 3.43y±1.91y |
| AL-ZOUBI A | 2015 | 16M/3F | 31.79±5.12 | 19T | 19A | 22.05m±9.96m |
| DEDA H | 2008 | 5M/4F | 28.11±9.40 | 6C,3T | 9A | 5.11y±5.32y |
| SABERI H | 2008 | 3M/1F | 35.75±9.91 | 4T | 2A,2C | 48.25m±22.63m |
| PARK HC | 2005 | 6M | 35.83±11.92 | 5C,1T | 6A | 7.83d±4.71d |
| BURGOS-GUTIÉRREZ C | 2022 | 128M/21F | 52.15±12.42 |  |  |  |
| SARASÚA JG | 2011 | 19M/3F | 54.61 |  |  |  |
| JEON SR | 2010 | 8M/2F | 46.00±8.51 | 10C | 4A,6B | 40.2m±40.3m |
| MIAO XY | 2015 |  |  |  |  |  |

This table summarizes the baseline characteristics from the involved articles. Missing values are due to insufficient data.

**Gender:** Males(M) and Females(F) of the trial. **Age:** Average age ± SD of the patients, or the age of the only patient. **Injury Site:** The injury segment(s) of the patients. Including Cervical (C), Thoracic (T), Lumbar (L) or Sacral (S). **Severity:** Severity of spinal cord injury according to AIS grading. **Duration:** The average time between the occurrance of spinal cord injury to start of treatment. Presented as Average time ± SD. Due to the inaccurate original data of some articles, interval of duration are provided instead. The following units are used: years(y), months(m), weeks(w), days(d).

**Supplementary Table 5:** Summary of Immunosuppressive Regimens and Related Adverse Events in Clinical Trials of Cell Therapy for Spinal Cord Injury

| **First Author** | **Year** | **Cell Origin** | **Cell Type** | **Immunosuppresor Usage** | **Drug** | **SAEs and AEs Related to Immunosuppression** |
| --- | --- | --- | --- | --- | --- | --- |
| CURTIS E | 2018 | allogeneic | neural stem cell | Yes | Mycophenolate mofeil+Tacrolimus | No AEs/SAEs |
| FESSLER RG | 2022 | allogeneic | oligodendrocyte progenitor cell | Yes | Tacrolimus | No SAEs; AEs:Hypomagnesemia+Nausea+Bacterial Infection |
| CHEN L | 2015 | allogeneic | olfactory ensheathing cell,Schwann cell | No |  |  |
| AL KANDARI S | 2018 | autologous and allogeneic |  | No |  |  |
| LIU J | 2013 | allogeneic | mesenchymal stromal cell | No |  |  |
| ALBU S | 2021 | allogeneic | mesenchymal stromal cell | No |  |  |
| WU J | 2012 | allogeneic | olfactory ensheathing cell | No |  |  |
| CHENG HB | 2014 | allogeneic | mesenchymal stromal cell | No |  |  |
| LEVI AD | 2018 | allogeneic | neural stem cell | Yes | Tacrolimus+Trimethoprim/sulfamethoxazole+Mycophenolate mofetil+Dexamethasone+Pantoprazole | SAEs: Sepsis |
| ZHAO YN | 2017 | allogeneic | mesenchymal stromal cell, scaffold | No |  |  |
| SHIN JC | 2015 | allogeneic | neural stem cell | Yes | Cyclosporin | No AEs/SAEs |
| DENG WS | 2020 | allogeneic | mesenchymal stromal cell, scaffold | No |  |  |
| DAI G | 2014 | allogeneic | mesenchymal stromal cell | No |  |  |
| RAO YJ | 2014 | allogeneic | olfactory ensheathing cell | No |  |  |
| LEVI AD | 2018 | allogeneic | neural stem cell | Yes | Unspecified | 6.4% of AEs/SAEs attributable to immunosuppression |
| SUGAI K | 2021 | allogeneic | iPSC-derived neural stem cell | Yes | Tacrolimus | Protocol Only |
| SHROFF G | 2016 | allogeneic | embryonic stem cell (multipotent stem cell) | No |  |  |
| GHOBRIAL GM | 2017 | allogeneic | neural stem cell | Yes | Unspecified | No AEs/SAEs |
| YAO LQ | 2013 | allogeneic | mononuclear cell | No |  |  |
| YANG Y | 2020 | allogeneic | mesenchymal stromal cell | No |  |  |
| MARTIN JR | 2025 | allogeneic | neural stem cell | Yes | Mycophenolate mofeil+Tacrolimus | No AEs/SAEs |
| TANG FW | 2021 | allogeneic | mesenchymal stromal cell, scaffold | No |  |  |
| HUANG HY | 2012 | allogeneic | olfactory ensheathing cell | No |  |  |
| RAO YJ | 2013 | allogeneic | olfactory ensheathing cell | No |  |  |
| KAPLAN N | 2025 | allogeneic | mesenchymal stromal cell | No |  |  |
| ZHU H | 2016 | allogeneic | mononuclear cell | No |  |  |
| YANG Y | 2021 | allogeneic | mesenchymal stromal cell | No |  |  |
| KODA M | 2024 | allogeneic | Muse cell (multipotent stem cell) | No |  |  |
| PAN K | 2019 | allogeneic | mesenchymal stromal cell | No |  |  |
| AKHLAGHPASAND M | 2024 | allogeneic | exosomes | No |  |  |
| AWIDI A | 2024 | autologous, allogeneic | mesenchymal stromal cell | No |  |  |
| YANG WZ | 2010 | allogeneic | mesenchymal stromal cell | No |  |  |
| XIAO ZF | 2018 | allogeneic | mesenchymal stromal cell, scaffold | No |  |  |
| ZHAO Y | 2021 | allogeneic | mesenchymal stromal cell | No |  |  |
| SMIRNOV VA | 2022 | allogeneic | mononuclear cell | No |  |  |
| MCKENNA SL | 2022 | allogeneic | oligodendrocyte progenitor cell | Yes | Tacrolimus | No SAEs; AEs:Hypomagnesemia+Nausea+Bacterial infection |
| CURT A | 2020 | allogeneic | neural stem cell | Yes | Tacrolimus+Mycophenolate mofetil+Dexamethasone | No SAEs;  AEs:Headache+Increased WBC+Unspecified mild AEs |
| MIAO XY | 2015 | allogeneic | mesenchymal stromal cell | No |  |  |

This table summarizes the baseline characteristics from the involved articles. Missing values are due to insufficient data.

**AEs:** Adverse Events; **SAEs:** Serious Adverse Events; **NSC:** Neural Stem Cell; **OPC:** Oligodendrocyte Progenitor Cell; **MSC:** Mesenchymal Stromal Cell; **OEC:** Olfactory Ensheathing Cell; **iPSC:** induced Pluripotent Stem Cell; **Muse cell:** Multilineage-differentiating Stress Enduring cell; **WBC:** White Blood Cell.

**Supplementary Figure 1. Influence of Administration Routes on the Safety Profile of Mesenchymal Stromal Cell (MSC) Therapies.**

Dumbbell plot illustrating the comparative incidence rates of specific adverse events stratified by delivery route exclusively within the MSC subgroup.

Abbreviations: MSC, mesenchymal stromal cell; IT, intrathecal; IV_IA, intravenous/intra-arterial; IL, intralesional; Combined, combined administration routes.

**Supplementary Figure 2. Distribution of Cell Dosages Stratified by Immunosuppression Status in Allogeneic Cell Therapy Trials.**

(A) Dosage distribution among allogeneic trials utilizing neural stem cells (NSCs; n=10), all of which employed concurrent immunosuppressive regimens.
(B) Dosage distribution among allogeneic trials utilizing non-NSC lineages (n=28), none of which utilized exogenous immunosuppression.

In both panels, "Others" designates trials where specific cell dosages were not explicitly reported.
